# LncRNA SOX2OT promotes temozolomide resistance by elevating SOX2 expression via ALKBH5-mediated epigenetic regulation in glioblastoma

**DOI:** 10.1038/s41419-020-2540-y

**Published:** 2020-05-21

**Authors:** Boyang Liu, Jian Zhou, Chenyang Wang, Yajie Chi, Quantang Wei, Zhao Fu, Changlin Lian, Qiongzhen Huang, Chenxin Liao, Zhao Yang, Huijun Zeng, Ningbo Xu, Hongbo Guo

**Affiliations:** 1 Department of Neurosurgery, The National Key Clinical Specialty, The Engineering Technology Research Center of Education Ministry of China, Guangdong Provincial Key Laboratory on Brain Function Repair and Regeneration, Zhujiang Hospital, Southern Medical University, Guangzhou, 510282 People’s Republic of China; 2 Department of Neurosurgery, Shunde Hospital, Southern Medical University (The First People’s Hospital of Shunde), Foshan, 528300 China; 3 Department of Neurosurgery, The First Affiliated Hospital of Shantou University Medical College, Shantou, 515041 China

**Keywords:** CNS cancer, Cell biology, Molecular biology

## Abstract

Temozolomide (TMZ) resistance is a major cause of recurrence and poor prognosis in glioblastoma (GBM). Recently, increasing evidences suggested that long noncoding RNAs (LncRNAs) modulate GBM biological processes, especially in resistance to chemotherapy, but their role in TMZ chemoresistance has not been fully illuminated. Here, we found that LncRNA SOX2OT was increased in TMZ-resistant cells and recurrent GBM patient samples, and abnormal expression was correlated with high risk of relapse and poor prognosis. Knockdown of SOX2OT suppressed cell proliferation, facilitated cell apoptosis, and enhanced TMZ sensitivity. In addition, we identified that SOX2OT regulated TMZ sensitivity by increasing SOX2 expression and further activating the Wnt5a/β-catenin signaling pathway in vitro and in vivo. Mechanistically, further investigation revealed that SOX2OT recruited ALKBH5, which binds with SOX2, demethylating the SOX2 transcript, leading to enhanced SOX2 expression. Together, these results demonstrated that LncRNA SOX2OT inhibited cell apoptosis, promoted cell proliferation, and TMZ resistance by upregulating SOX2 expression, which activated the Wnt5a/β-catenin signaling pathway. Our findings indicate that LncRNA SOX2OT may serve as a novel biomarker for GBM prognosis and act as a therapeutic target for TMZ treatment.

## Introduction

Glioblastoma^1^ (GBM) is the most malignant intracranial tumor^2^, accounting for over 50% of primary brain tumors, with median survival time of <1 year with high relapse and poor prognosis^3^. Standard clinical treatment is surgical resection and postoperative radiotherapy and chemotherapy^4^. Temozolomide (TMZ), an oral alkylating agent, with 100% bioavailability passes the blood–brain barrier and has few side effects, is the first line chemotherapy drug after surgical excision^5^. However, TMZ resistance often occurs in patients in the middle and late stages of chemotherapy, which severely restricts its therapeutic efficacy and leads to treatment failure^6^. TMZ resistance is likely to involve multiple factors and various mechanisms. At present, these mechanisms are thought to include DNA damage repair, such as source chromosome recombination repair, O^6^-methylguanine-DNA-methyltransferase (MGMT) repair; ectopic expression of multi-drug resistance related proteins (MRPs); abnormal apoptosis pathways; the presence of tumor stem cells; and changes in tumor immune microenvironment, like increased protective autophagy and non-folding protein reactions^7,8^. Of these, MGMT is considered the main molecule, leading to TMZ resistance in GBM^9,10^. However, because some patients with low expression of MGMT still acquire resistance to TMZ, other mechanisms involved in TMZ resistance, are also likely to be important.

Long noncoding RNAs (LncRNAs) are a novel class of RNAs of over 200 nucleotides without no protein-coding ability, but have important biological functions involving transcription, post-transcription, and epigenetics^11^. LncRNAs can regulate tumor genesis via alternative splicing, chromatin recruitment, and epigenetic modification mechanisms, and are expected to be a new target for tumor diagnosis and treatment^12^. Various LncRNAs are closely related to tumor chemoresistance in hepatocellular carcinoma^13^, and ovarian cancer^14^. LncRNAs have also been shown to be involved in TMZ resistance^15,16^. Thus, targeting dysregulated LncRNAs may provide an alternative therapeutic strategy for TMZ resistance. Previously, we screened differentially expressed LncRNAs through microarray in TMZ-resistant cells^17^. Several LncRNAs related to TMZ resistance were found, but LncRNA SOX2OT and its nearby gene, sex determining region Y-box 2 (SOX2), were of note because they were upregulated in TMZ-resistant cells, and previous studies demonstrated that SOX2OT participates in genesis of various malignant tumors by regulating different molecules^18^. However, far less known about its role in regulation of TMZ resistance as well as the underlying mechanisms.

It is known that LncRNAs exert a critical biological role by regulating the expression of proximal genes^19^. Therefore, the significantly high level of SOX2 in TMZ-resistant cells may result from overexpression of SOX2OT. SOX2 is a transcription factor containing high mobility group (HMG) domain and is an important member of the highly conserved SOX family. SOX2 has an important role in regulating early embryonic and normal tissue development, maintaining pluripotency of stem cells and determining cell fate^20,21^. SOX2 is also a potential carcinogenic factor associated with malignancy, lymph node metastasis, and pathological grading and clinical staging, especially in glioma cells, it has a crucial role in maintaining stem cell characteristics^22,23^. Moreover, SOX2 is closely related to tumor chemoresistance. SOX2 can activate the Wnt/β-catenin signaling pathway and participate in cisplatin resistance of lung adenocarcinoma^24^, and via MCAM can promote chemotherapy resistance in small cell lung cancer^25^. Previous studies have also shown that SOX2OT can specifically regulate SOX2 to promote cell proliferation in other tumors^26^, and it can also regulate miR-194-5p and miR-122 to maintain the stem cell characteristics in glioma cells^27^. However, whether SOX2 is the potential target of SOX2OT in TMZ resistance remains unclear and needs to be further confirmed and studied.

In this study, we hypothesized that SOX2OT increased SOX2 expression, resulting in acquired TMZ resistance. To verify this hypothesis, we first detected the expression of SOX2OT in glioma tissues and cell lines and evaluated its clinical relevance. Next, we explored the role of SOX2OT and its potential targeted gene SOX2 on cell growth and apoptosis by performing in vitro and in vivo experimental assays. Finally, we verified SOX2OT–SOX2 binding of targets and the underlying mechanisms. To our knowledge, we report the function of SOX2OT in TMZ resistance for the first time. The results of this study suggest a new potential underlying molecular target to reverse TMZ chemoresistance in GBM.

## Results

### Elevated LncRNA SOX2OT expression is associated with TMZ resistance and poor prognosis in GBM

**Fig. 1:**
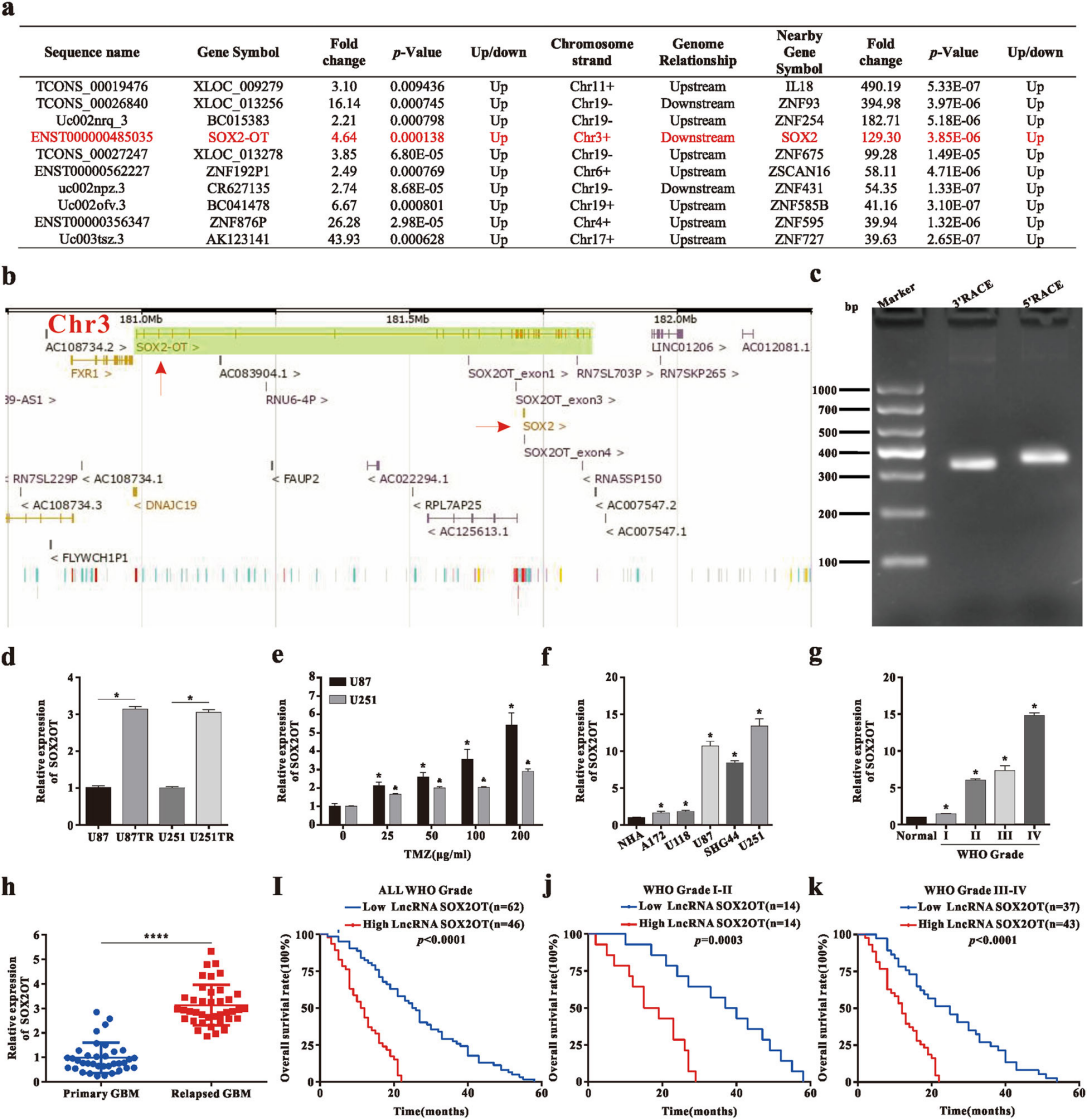
Elevated LncRNA SOX2OT expression is associated with TMZ resistance and poor prognosis in GBM. **a** Ten significantly upregulated LncRNAs and mRNA expression of their nearby genes in LncRNAs microarray. **b** SOX2OT locates next to the SOX2 gene on human chromosome 3. SOX2OT is encoded by the (+) DNA strand, and SOX2 is also coded by the (+) DNA strand. **c** 5’ RACE (rapid amplification of cDNA ends) and 3’ RACE of SOX2OT in GBM cells. **d** SOX2OT expression levels in TMZ-resistant and its parental GBM cells, *p < 0.05 compared with U87 or U251 cells. **e** SOX2OT expression levels in U87 or U251 cells under different concentrations TMZ treatment, *p < 0.05 and ^&^*p* < 0.05 compared with U87 and U251 cells, respectively. **f** SOX2OT expression levels in various glioma cell lines, compared with the normal human astrocytes (NHA), *p* < 0.05 compared with NHA cells. **g** The qRT-PCR analysis of SOX2OT expression in GBM tissues with different tumor grades (*n* = 3), normal (*n* = 3), *p < 0.05 compared with the normal samples. **h** SOX2OT expression levels in primary and relapsed GBM tissues were examined by qRT-PCR and normalized against U6 expression, primary GBM (*n* = 36), relapsed GBM (*n* = 39), ****p < 0.0001 compared with primary GBM tissues. **i**–**k** The patients were classified into two groups according to SOX2OT expression level, which was detected via qRT-PCR analysis, and the median value for glioma cases was chosen as the cutoff point with which the cases were separated into high and low SOX2OT level group; Kaplan–Meier overall survival curves according to SOX2OT expression level. Data are presented as mean ± SD. of three independent experiments.

Microarrays of LncRNAs and mRNAs in the U87TR and U87 showed SOX2OT and SOX2 were both upregulated in TMZ-resistant cells^17^, especially, the expression of SOX2 was increased 129.30-fold (Fig. 1a). As LncRNAs have been reported to either positively or negatively regulate neighboring genes, the genomic locations were characterized. SOX2OT (ENST00000485035.1) was located ~12 kb upstream of the SOX2 gene (Fig. 1b) (Ensembl Release 98. http://asia.ensembl.org/index.html). The 5’ RACE and 3’ RACE demonstrated that the length of this transcript was 583 base pairs (bp) (Fig. 1c). To further investigate whether SOX2OT was associated with TMZ chemoresistance, we performed qRT-PCR analysis, and the results showed that SOX2OT expression was markedly increased in resistant cells, as compared with parental cells (Fig. 1d). Furthermore, SOX2OT expression levels were upregulated in a dose-dependent manner (Fig. 1e). Expression of SOX2OT in various cell lines showed its expression level was higher in glioma cell lines than in normal human astrocytes (NHA) (Fig. 1f). Expression of SOX2OT in 118 surgical specimens from glioma patients who were receiving TMZ chemotherapy (Supplementary Table S1) showed that SOX2OT level was positively associated with tumor grading (WHO I/II versus WHO III/IV) (*p* < 0.001), confirmed by qRT-PCR analysis (Fig. 1g). SOX2OT expression in relapsed GBM patients with TMZ chemotherapy was higher than that in primary GBM patients (Fig. 1h). In addition, Kaplan–Meier analysis showed that glioma patients with higher SOX2OT expression obtained shorter median survival time and poor prognosis when compared with the patients with lower expression (Fig. 1i–k). These data revealed that SOX2OT upregulation correlated to TMZ chemoresistance and poor prognosis in GBM.

### SOX2OT upregulation confers TMZ resistance, facilitates cell proliferation, and inhibits cell apoptosis in GBM cells

**Fig. 2:**
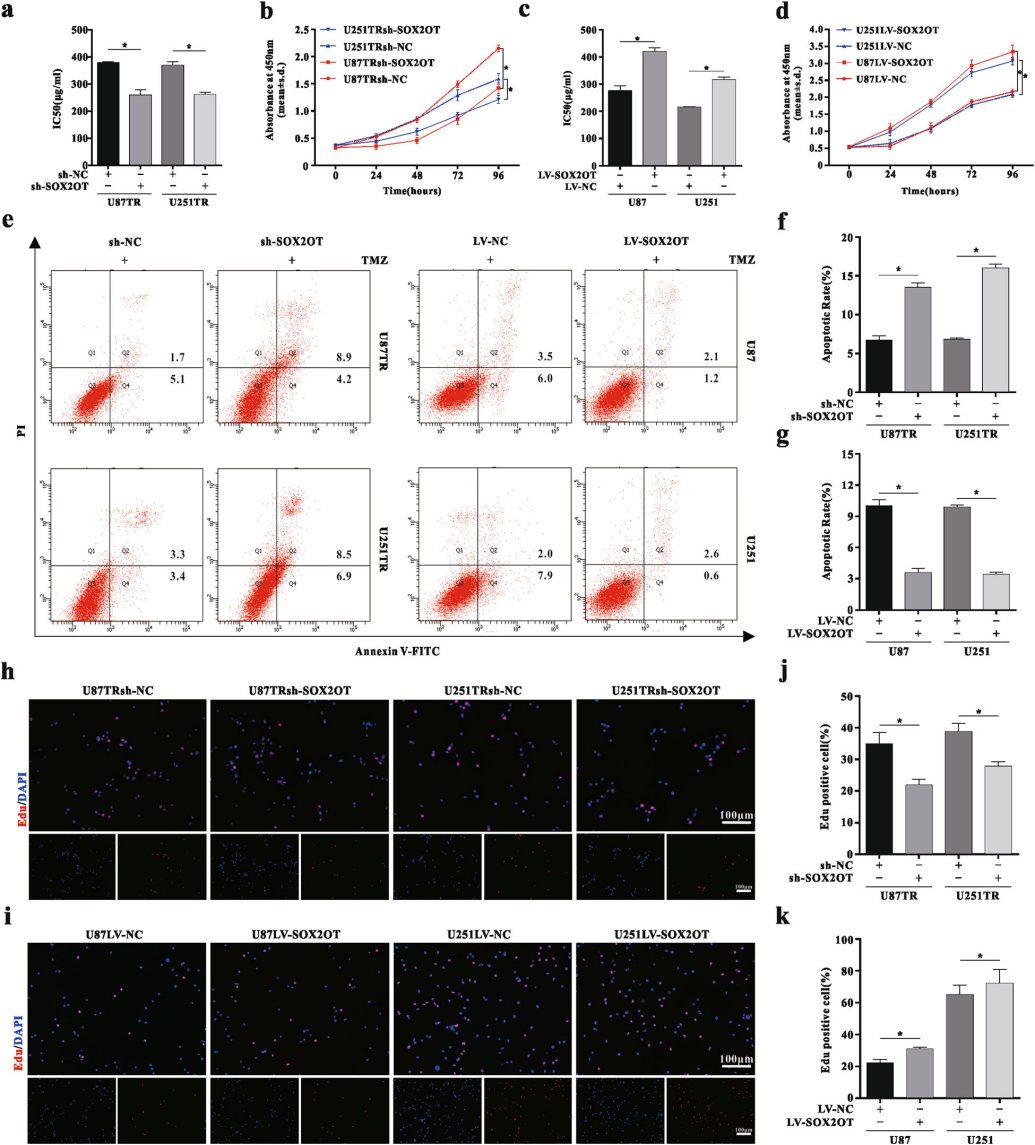
SOX2OT upregulation confers TMZ resistance, facilitates cell proliferation, and inhibits cell apoptosis in GBM cells. **a** The TMZ sensitivity of U87TR and U251TR cells after sh-NC or sh-SOX2OT transfection measured by CCK-8 assay. *p < 0.05 compared with sh-NC group cells. **b** Cell viability of U87TR and U251TR sh-NC or sh-SOX2OT cells treated with TMZ (50 μg/ml) for 24, 48, 72, and 96 h. *p < 0.05 compared with sh-NC group cells. **c** The TMZ sensitivity of U87 and U251 cells after LV-NC or LV-SOX2OT transfection measured by CCK-8 assay. *p < 0.05 compared with LV-NC group cells. **d** Cell viability of U87 and U251 LV-NC or LV-SOX2OT cells treated TMZ (50 μg/ml) for 24, 48, 72, and 96 h. *p < 0.05 compared with LV-NC group cells. **e**–**g** The rate of apoptosis of sh-SOX2OT or LV-SOX2OT cells treated with TMZ (50 μg/ml) for 48 h was determined by the Annexin-V/FITC double staining. *p < 0.05 compared with sh-NC or LV-NC group cells. **h**–**k** The rate of EdU cells in sh-SOX2OT or LV-SOX2OT cells treated with TMZ (50 μg/ml) for 48 h was determined by EdU staining. *p < 0.05 compared with sh-NC or LV-NC group cells. Scar bar = 100 μm. Data are presented as mean ± S.D. of three independent experiments.

Cells were transfected with sh-NC or sh-SOX2OT lentiviral vectors to knockdown SOX2OT expression and its efficiency was confirmed by qRT-PCR analysis (Fig. S1a). Stable SOX2OT overexpressed cells were made by transfection with LV-NC or LV-SOX2OT lentiviral vectors, respectively (Fig. S1b). CCK-8 assay showed loss of SOX2OT significantly decreased cell viability upon TMZ (50 μg/ml) treatment and chemoresistance, resulting in lower IC_50_ values (Fig. 2a,b, and S1c). In contrast, overexpressed SOX2OT increased cell viability with higher IC_50_ values (Fig. 2c,d, and S1d). Furthermore, the flow cytometry (FCM) assay showed that knockdown of SOX2OT increased apoptosis, whereas overexpression of SOX2OT decreased apoptosis (Fig. 2e–g and S1e–g). In addition, we found a positive correlation between SOX2OT and MRPs, including BCRP1, MDR1, MRP1, SOX2OT promoted the expression of these proteins (Fig. S1h–m). Overexpressed SOX2OT also increased levels of pluripotent transcription factors related to tumor chemoresistance, whereas knocking down its expression presented the opposite effect (Fig. S1n–q). The percentage of the EdU cells significantly decreased in sh-SOX2OT groups and increased in LV-SOX2OT groups (Fig. 2h–k). These data indicate that SOX2OT confers TMZ chemoresistance, facilitates cell proliferation and inhibits cell apoptosis in TMZ-resistant cells.

### SOX2 is a downstream target of SOX2OT in GBM cells

**Fig. 3:**
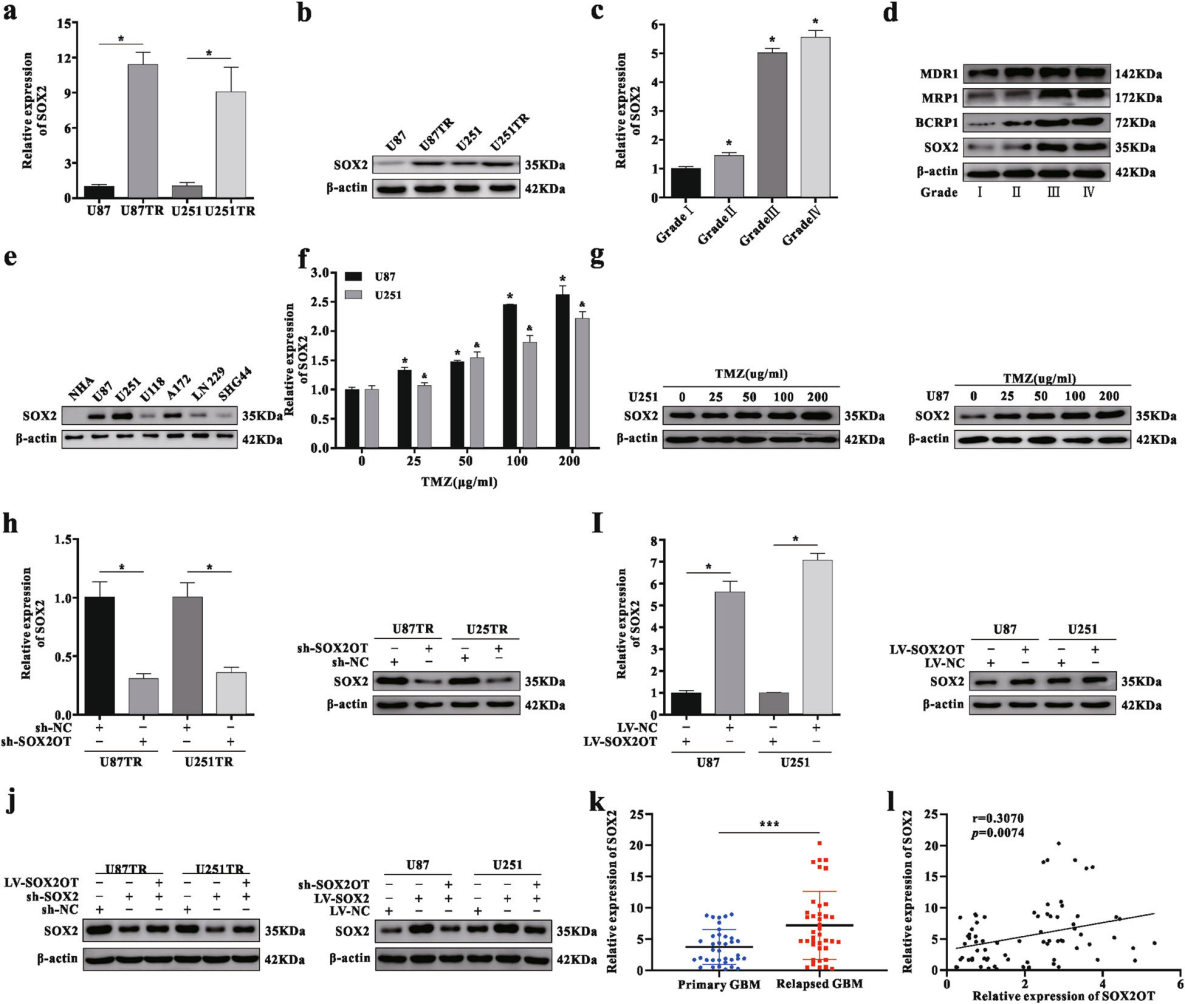
SOX2 is a downstream target of SOX2OT in GBM cells. **a**–**b** The mRNA and protein expression of SOX2 in TMZ-resistant and its parental cells, *p < 0.05 compared with U87 or U251 cells. **c** The mRNA expression of SOX2 in different grades gliomas (*n* = 3), *p < 0.05 compared with the GBM grade I samples. **d** The protein levels of SOX2 and multi-drug-related genes in various grade gliomas (*n* = 3). **e** The protein expression of SOX2 in various glioma cell lines was detected via western blot analysis, compared with the normal human astrocytes (NHA). **f**–**g** The SOX2 expression levels in U87 or U251 cells under different TMZ concentrations was measured through qRT-PCR and western blot assay. *p < 0.05 or ^&^*p* < 0.05 compared with no TMZ groups. **h**–**i** The SOX2 expression was detected via qRT-PCR or western blot assay after transfection with sh-SOX2OT or LV-SOX2OT lentiviral vectors, *p < 0.05 compared with sh-NC or LV-NC group. **j** Co-transfection of LV-SOX2OT reversed the protein level of SOX2 knockdown, whereas co-expression of sh-SOX2OT abrogated the effects induced by LV-SOX2 in GBM cells. **k** SOX2 expression levels in primary and relapsed GBM tissues were examined by qRT-PCR and normalized against U6 expression, primary GBM (*n* = 36), relapsed GBM (*n* = 39), ****p < 0.0001 compared with primary GBM tissues. **l** The relationship of SOX2OT and SOX2 expression in 75 GBM tissues through qRT-PCR analysis.

We verified that the expression of SOX2 was markedly elevated at both mRNA and proteins levels (Fig. 3a, b). Similarly, SOX2 expression levels were increased in higher grade gliomas compared with Grade I samples, consistent with the expression trend of MRPs (Fig. 3c, d), and the expression level was higher in the glioma cell lines when compared with NHA (Fig. 3e). Furthermore, SOX2 expression levels were also detected under different TMZ treatment concentrations, and the results indicated that the expression levels were increased in a dose-dependent manner (Fig. 3f–g). Finally, knockdown of SOX2OT led to decreased expression of SOX2, whereas overexpression of SOX2OT increased SOX2 expression (Fig. 3h–i). Similarly, SOX2OT reversed the SOX2 expression in co-transfected cells (Fig. 3j). SOX2 expression in relapsed GBM patients with TMZ chemotherapy was higher than that in primary GBM patients (Fig. 3k), and a positive correlation was noted between SOX2OT and SOX2 (Fig. 3l). These results strongly indicate that SOX2 serves as a target gene of SOX2OT in glioma.

### SOX2OT binds with RNA demethylase ALKBH5, which is involved in TMZ resistance

**Fig. 4:**
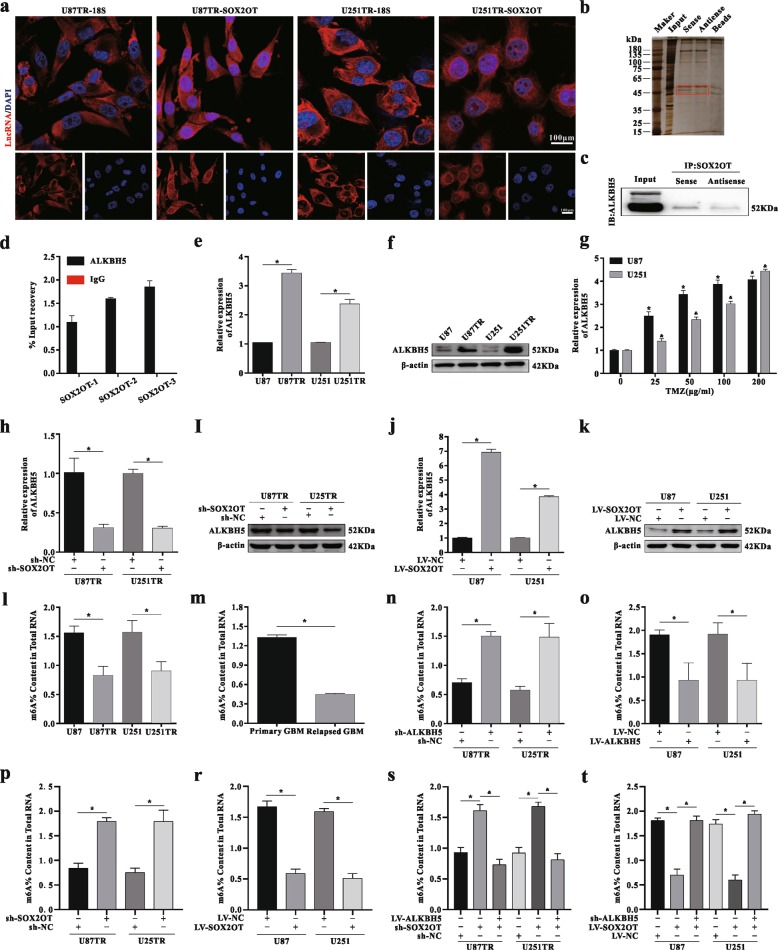
SOX2OT binds with RNA demethylase ALKBH5, which is involved in TMZ resistance. **a** Cellular localization of SOX2OT in U87TR and U251TR cells by RNA FISH assay. Scar bar = 100 μm. **b**–**c** RNA pulldown assay and western blot were performed to verify the enrichment of ALKBH5 by LncRNA SOX2OT in GBM cells. **d** RIP-qPCR assay was used to verify that ALKBH5 binds with LncRNA SOX2OT with three special primers, *p < 0.05 compared with the IgG group. **e**–**f** Expression of ALKBH5 in TMZ resistant and its parental cells was measured via qRT-PCR assay. *p < 0.05 compared with U87 or U251 cells. **g** Expression of ALKBH5 under different TMZ concentrations was measured by qRT-PCR analysis, *p < 0.05 or ^&^*p* < 0.05 compared with no TMZ grou*p*s. **h**–**k** The ALKBH5 expression was detected via qRT**-**PCR or western blot assay after transfected with sh-SOX2OT or LV-SOX2OT lentiviral vectors, *p < 0.05 compared with sh-SOX2OT or LV-SOX2OT group. **l** The m^6^A levels of total RNAs in TMZ-resistant and its parental cells were detected via m^6^A quantitative assay. *p < 0.05 compared with U87 or U251 cells. **m** The m^6^A levels of total RNAs in GBM tissues were detected by m^6^A quantitative kit. *p < 0.05 compared with the primary GBM. **n**–**o** The m^6^A levels in sh-ALKBH5 or LV-ALKBH5 transfected cells were identified. *p < 0.05 compared with sh-NC or LV-NC group. **p**–**r** The m^6^A levels in sh-SOX2OT or LV-SOX2OT transfected cells were identified. *p < 0.05 compared with sh-NC or LV-NC group. **s** The m^6^A levels in sh-SOX2OT and LV-ALKBH5 co-transfected cells were identified. *p < 0.05 compared with sh-SOX2OT group. **t** The m^6^A levels in LV-SOX2OT and sh-ALKBH5 co-transfected cells were identified. *p < 0.05 compared with LV**-**SOX2OT group.

Specific SOX2OT probes were designed and used in an RNA FISH assay, and the results indicated that SOX2OT was both in the cytoplasm and nucleus in GBM cells (Figs. 4a, and S2a). Therefore, we speculated that SOX2OT could function as a scaffold to recruit RNA-binding proteins (RBPs), regulating downstream targeted gene expression. To clarify this hypothesis, we searched the LncRNAtor database (The LncRNAtor. http://lncrnator.ewha.ac.kr/index.htm) to predict potential RBPs for SOX2OT. Bioinformatic analysis showed that ALKBH5, CCNT1, CPSF100, DGCR8, and HuR may bind with SOX2OT (Fig. S2b). Then RNA pulldown and RIP-qPCR results demonstrated that ALKBH5 could combine with SOX2OT (Fig. 4b–d). ALKBH5 expression was markedly upregulated in TMZ-resistant cells compared with their parental cells (Figs. 4e–f and S2c–d). Moreover, the expression levels increased in a dose-dependent style under upon different TMZ treatment concentrations (Figs. 4g and S2e–f). ALKBH5 expression was significantly downregulated after SOX2OT knockdown, whereas its expression was increased in LV-SOX2OT transfected cells (Fig. 4h–k). To clarify whether overexpressed ALKBH5 decreased m^6^A levels in TMZ-resistant cells and clinical tissue samples, a m^6^A quantitative assay was used. The results showed that the m^6^A level in TMZ-resistant cells was downregulated compared with that in parental cells (Fig. 4l). Meanwhile, the amount of m^6^A in recurrent tissues was lower than that of the primary samples (Fig. 4m). Finally, we measured the changes of m^6^A levels in cells transfected with sh-ALKBH5 or LV-ALKBH5 lentiviral vectors. The knockdown or overexpression efficiency was confirmed by qRT-PCR and western blot analysis (Fig. S2g–j). As we expected, the m^6^A level of total RNAs increased in sh-ALKBH5 transfected cells compared with sh-NC group (Fig. 4n), whereas overexpressed ALKBH5 decreased the m^6^A levels (Fig. 4o). Furthermore, the m^6^A level of total RNAs increased in sh-SOX2OT-transfected cells compared with the sh-NC group (Fig. 4p), whereas overexpressed SOX2OT decreased the m^6^A levels in GBM cells (Fig. 4r). In co-transfected cells, ALKBH5 could reverse the change in m^6^A levels caused by sh-SOX2OT or LV-SOX2OT transfection (Fig. 4s–t). Taken together, these results suggest SOX2OT binds with the RNA demethylase ALKBH5, which mediates m^6^A levels in TMZ-resistant cells.

### ALKBH5 maintains SOX2 expression by demethylating SOX2 transcripts

**Fig. 5:**
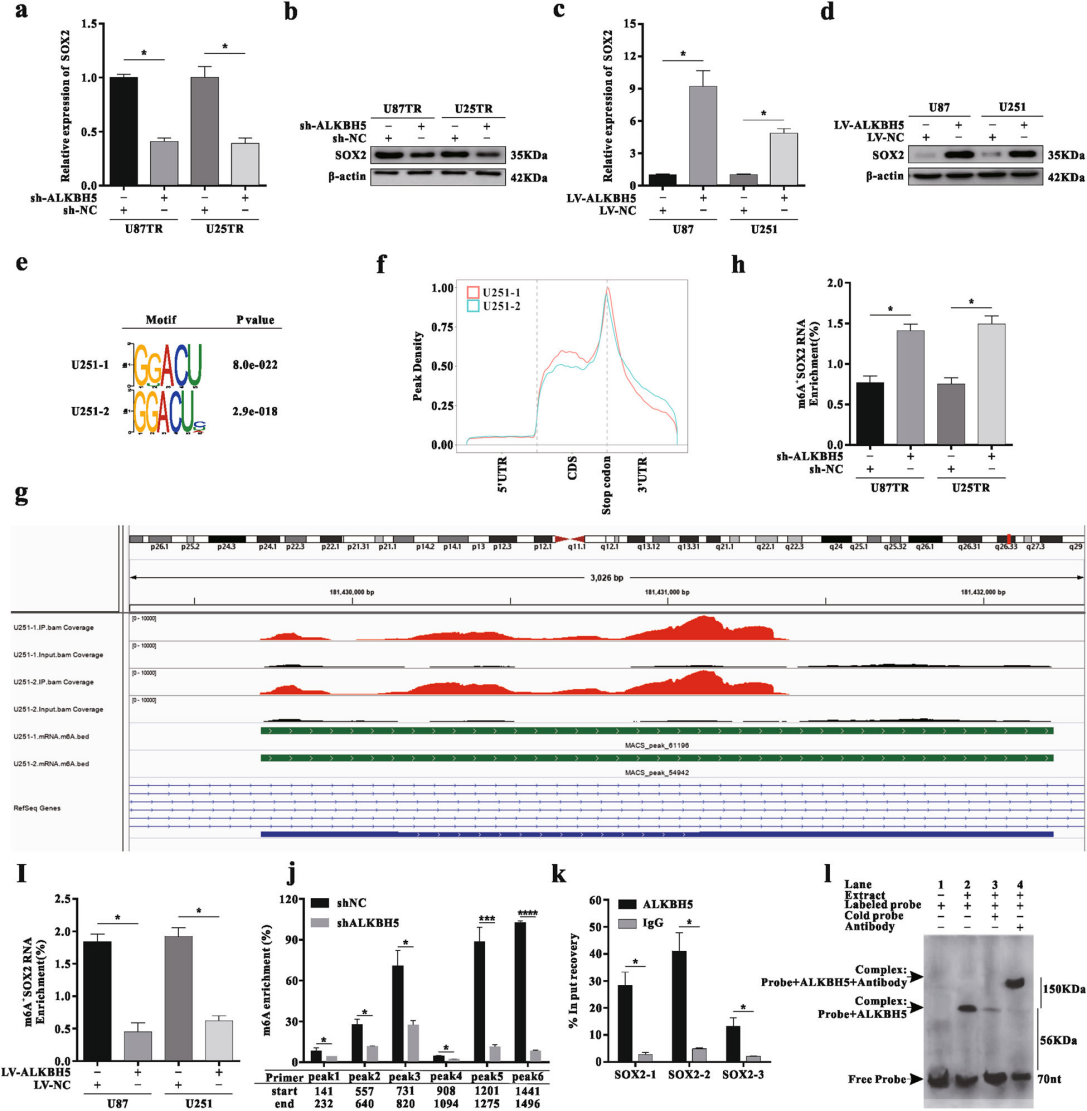
ALKBH5 maintains SOX2 expression by demethylating SOX2 transcripts. **a**–**b** The SOX2 expression was detected via qRT-PCR or western blot assay after transfection with sh-ALKBH5 lentiviral vectors, *p < 0.05 compared with sh-NC group. **c**–**d** The SOX2 expression was detected via qRT-PCR or western blot assay after transfection with LV-ALKBH5 lentiviral vectors, *p < 0.05 compared with LV-NC group. **e** Sequence motif identified with m^6^A-seq peaks in GBM cells. **f** Distribution of m^6^A peaks reads across all mRNAs. **g** The visual peaks figure of relative abundance of m^6^A sites along SOX2 mRNA in GBM cells. **h**–**i** MeRIP-qPCR analysis of intact SOX2 RNA from GBM cells with sh-ALKBH5 transfected or LV-ALKBH5 transfected, *p < 0.05 compared with sh-NC or LV-NC group. **j** MeRIP-PCR analysis of fragmented SOX2 RNA in GBM cells with or without ALKBH5 knockdown, ****p < 0.0001 compared with sh-NC grou*p*. **k** The qPCR analysis of SOX2 enrichment in chromatin immunoprecipitation, *p < 0.05 compared with the IgG group. **l** RNA EMSA analysis of ALKBH5 binding to the CpG region of SOX2 gene.

ALKBH5 knockdown markedly decreased the mRNA and protein expression of SOX2 (Fig. 5a–b). In contrast, overexpressed ALKBH5 exerted the opposite effect (Fig. 5c–d). m^6^A sequencing (m^6^A-seq) showed the GGACU motif was highly enriched within m^6^A sites GBM cells (Fig. 5e). A metagene analysis revealed that m^6^A peaks were especially abundant in the vicinity of stop codons, with a subset of m^6^A peaks located in the internal exons (Fig. 5f). Four significant m^6^A peaks were identified in SOX2 mRNA, indicating that ALKBH5 may regulate SOX2 expression via RNA demethylation (Fig. 5g). Methylated RNA immunoprecipitation (MeRIP) combined with qRT-PCR showed the m^6^A level was significantly elevated in sh-ALKBH5 cells, whereas lower m^6^A content was detected in LV-ALKBH5-transfected cells (Fig. 5h–i). Using specific primers to detect each peak region, we demonstrated that m^6^A enrichment of peak 4 was elevated one- to twofold in sh-ALKBH5 cells (Fig. 5j). Moreover, a ChIP-qPCR assay indicated that ALKBH5 bound with the promoter region of the SOX2 transcript (Fig. 5k). An RNA electrophoretic mobility-shift assay (EMSA) demonstrated that ALKBH5 bound to the CpG area of the SOX2 upstream transcript, forming a large complex when mixed with an ALKBH5 antibody (Fig. 5l). Collectively, these data suggested that ALKBH5 binds to the promoter region of the SOX2 gene, and increased SOX2 expression through suppressing its m^6^A methylation level.

### SOX2OT regulates TMZ resistance through SOX2

**Fig. 6:**
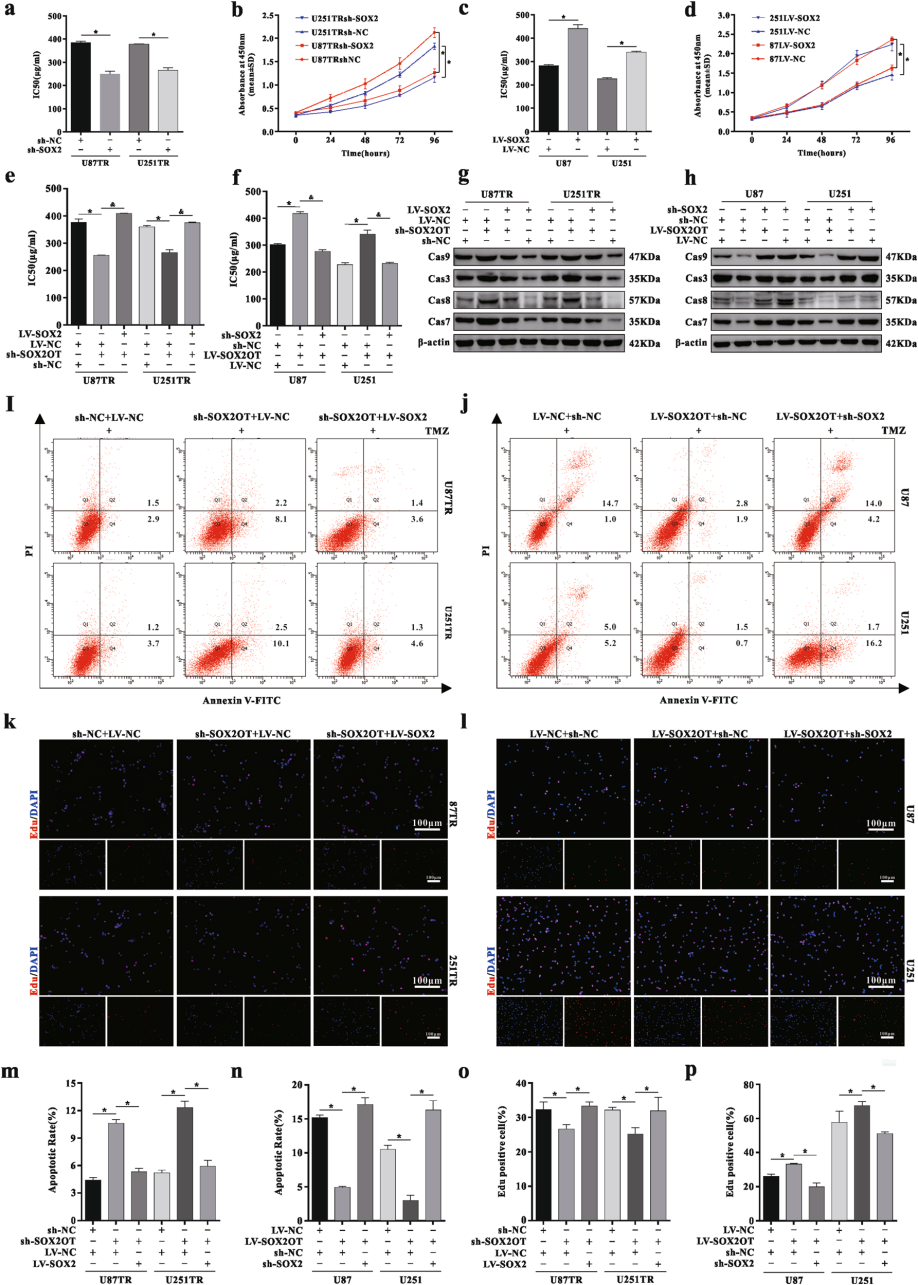
SOX2OT regulates TMZ resistance through SOX2. **a** The TMZ sensitivity of U87TR and U251TR cells upon sh-NC or sh-SOX2 transfected through CCK-8 assay. *p < 0.05 compared with sh-NC group cells. **b** Cell viability of U87TR and U251TR sh-NC or sh-SOX2 transfected cells treated with TMZ (50 μg/ml) for 24, 48, 72, and 96 h. *p < 0.05 compared with sh-NC group cells. **c** The TMZ sensitivity of U87 and U251 cells upon LV-SOX2 transfected measured by CCK-8 assay. *p < 0.05 compared with LV-NC group cells. **d** Cell viability of U87 and U251 LV-NC or LV-SOX2 cells treated with TMZ (50 μg/ml) for 24, 48, 72, and 96 h. *p < 0.05 compared with LV-NC group cells. **e** The TMZ sensitivity of resistant cells with sh-SOX2OT and LV-SOX2 co-transfection was measured through CCK-8 assay. *p < 0.05 compared with sh-SOX2OT and LV-SOX2 co-transfected group. **f** The TMZ sensitivity of parental cells with co-transfected LV-SOX2OT and sh-SOX2 was measured through CCK-8 assay. *p < 0.05 compared with LV-SOX2OT and sh-SOX2 co-transfected group. **g** The western blot analysis of apoptosis-related genes in sh-SOX2OT and LV-SOX2 co-transfected cells. **h** The western blot analysis of apoptosis-related genes in LV-SOX2OT and sh-SOX2 co-transfected cells. **i** The rate of apoptosis of U87TR and U251TR cells co-transfected with sh-SOX2OT and sh-SOX2 upon TMZ (50 μg/ml) treatment for 48 h measured by flow cytometry analysis. **j** The rate of apoptosis of U87 and U251 cells co-transfected with LV-SOX2OT and sh-SOX2 upon TMZ (50 μg/ml) treatment for 48 h measured by flow cytometry analysis. **k** The rate of Edu-positive cells in TMZ-resistant cells co-transfected with sh-SOX2OT and LV-SOX2 after treatment with TMZ (50 μg/ml) for 48 h determined by EdU staining assay. Scar bar = 50 μm. **l** The rate of Edu-positive cells in parental cells co-transfected with LV-SOX2OT and sh-SOX2 after treatment with TMZ (50 μg/ml) for 48 h determined by EdU staining assay. Scar bar = 50 μm. **m**–**p** The statistics of apoptosis rate and Edu-positive cell rate. *p < 0.05 compared with sh-SOX2OT and LV-SOX2 co-transfected group or LV-SOX2OT and sh-SOX2 co-transfected group. Data are presented as mean ± S.D. of three independent experiments.

First, to identify whether SOX2 was involved in TMZ resistance, sh-SOX2 lentiviral vectors and LV-SOX2OT lentiviral vectors were transfected (Fig. S3a–b). CCK-8 assay showed that silenced SOX2 significantly decreased cell viability, compared with the sh-NC group (Figs. 6a–b, and S3c). Inversely, overexpressed SOX2 exerted the opposite effect (Figs. 6c–d and S3d). As we shown, overexpressed SOX2 also increased the levels of MRPs, whereas knocking down exerted the opposite effect (Fig. S3e–j). Moreover, FCM analysis showed that inhibition of SOX2-induced apoptosis, whereas overexpression of SOX2 decreased apoptotic rate (Figs. S3o–q and S4a–c). In addition, the EdU assay also demonstrated that the percentage of the EdU cells was decreased in the sh-SOX2-transfected group (Fig. S4h). Although, the percentage of the EdU cells was increased after LV-SOX2 transfected (Fig. S4i). These data suggested that SOX2 promoted TMZ resistance by promoting cell proliferation and inhibiting cell apoptosis in GBM cells.

To further explore whether SOX2 was involved in TMZ resistance mediated by SOX2OT, LV-SOX2 lentiviral vectors were co-transfected into the sh-SOX2OT cells. CCK-8 assays showed that SOX2 overexpression markedly increased cell viability and promoted the SOX2OT-mediated chemoresistance (Figs. 6e and Fig. S3k–l). Inversely, SOX2 interference significantly decreased cell viability and suppressed TMZ resistance in LV-SOX2OT co-transfected cells (Figs. 6f and S3m–n). Western blot analysis revealed that SOX2 could reverse the changes in apoptosis-related protein expression level caused by SOX2OT (Fig. 6g–h). In addition, in FCM analysis, overexpressed SOX2 markedly suppressed apoptosis in sh-SOX2OT co-transfected cells, and SOX2 interference dramatically increased the apoptotic rate in LV-SOX2OT co-transfected cells (Figs. 6i–j, and S4d–f). Finally, the percentage of EdU-positive cells was significantly increased in the LV-SOX2 and sh-SOX2OT co-transfected group, whereas knockdown of SOX2 inhibited cell growth (Figs. 6kl, S4g–i). The statistics of apoptosis rate and Edu-positive cell rate are shown in the figure below (Fig. 6m–p). Together, these results suggested that SOX2OT regulated chemoresistance by upregulating SOX2 expression in GBM cells.

### The Wnt5a/β-catenin signaling pathway is involved in SOX2OT-regulating SOX2 in TMZ resistance

**Fig. 7:**
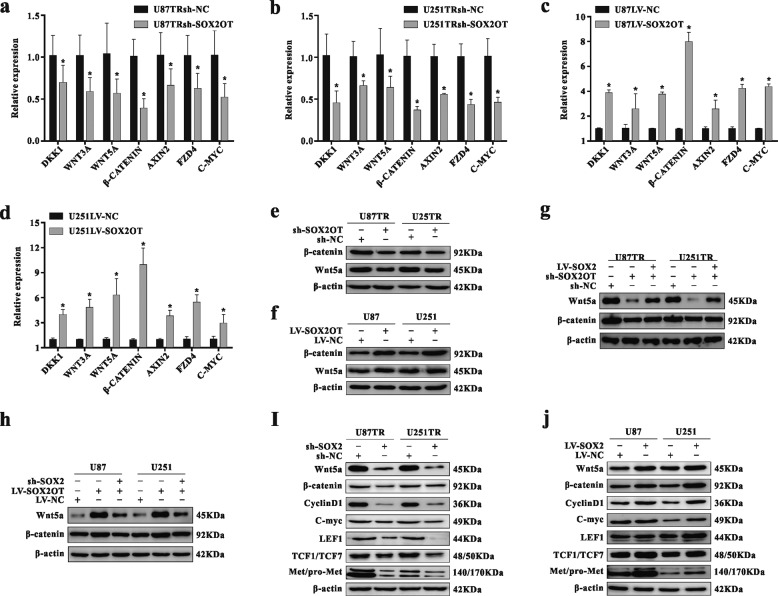
The Wnt5a/β-catenin signaling pathway is involved in SOX2OT-regulating SOX2 in TMZ resistance. **a**–**b** qRT-PCR analysis for mRNA expression of Wnt/β-catenin signaling pathway-related genes in TMZ-resistant cells after sh-NC or sh-SOX2OT transfection, *p < 0.05 compared with sh-SOX2OT group. **c**–**d** qRT**-**PCR analysis for mRNA expression of Wnt/β-catenin signaling pathway-related genes in parental cells with LV-NC or LV-SOX2OT transfection, *p < 0.05 compared with LV-SOX2OT group. **e**–**f** Western blot analysis for protein expression of β-catenin, Wnt5a in glioma cells with sh-SOX2OT or LV-SOX2OT transfection. **g** The protein levels of β-catenin, Wnt5a in U87TR and U251TR cells co-transfected with sh-SOX2OT and LV-SOX2. **h** The protein levels of β-catenin, Wnt5a in U87 and U251 cells co-transfected with LV-SOX2OT and sh-SOX2. **i** The protein levels of Wnt/β-catenin signaling pathway-related genes in sh-NC or sh-SOX2-transfected cells. **j** The protein levels of Wnt/β-catenin signaling pathway-related genes in LV-NC or LV-SOX2 transfected cells.

Analysis with qRT-PCR revealed that the Wnt/β-catenin signaling pathway was activated in TMZ-resistant cells compared with parental cells (Fig. S5a–b). We hypothesized that Wnt/β-catenin signaling pathway may be involved in the TMZ chemoresistance regulation medicated by the SOX2OT/SOX2 axis. Western blot and qRT-PCR found that Wnt5a/β-catenin activity was significantly suppressed in sh-SOX2OT-transfected cells (Fig. 7a–b, e), whereas ectopic expression of SOX2 reversed this effect (Fig. 7g). In contrast, Wnt5a/β-catenin activity was increased in LV-SOX2OT transfected group (Fig. 7c–d, f), whereas silencing of SOX2 diminished this enhanced activity (Fig. 7h). Moreover, expression levels of Wnt5a/β-catenin under different TMZ concentrations were increased in a dose-dependent manner (Fig. S5c–d). Furthermore, we found that knockdown of SOX2 expression inhibited the Wnt5a/β-catenin signaling pathway and significantly decreased expression of downstream target genes, such as CyclinD1, C-myc, LEF1, TCF1/7, and Met/pro-Met, which are related to cell proliferation and cell apoptosis (Fig. 7i). Conversely, ectopic expression of SOX2 promoted this effect (Fig. 7j). Finally, SOX2 reversed the changes in expression level of these proteins caused by SOX2OT (Fig. S5e–f). To this end, these results indicated that SOX2OT promoted cell growth by upregulating SOX2 expression and further activation of the Wnt5a/β-catenin signaling pathway.

### SOX2OT depletion enhances TMZ sensitivity in vivo

**Fig. 8:**
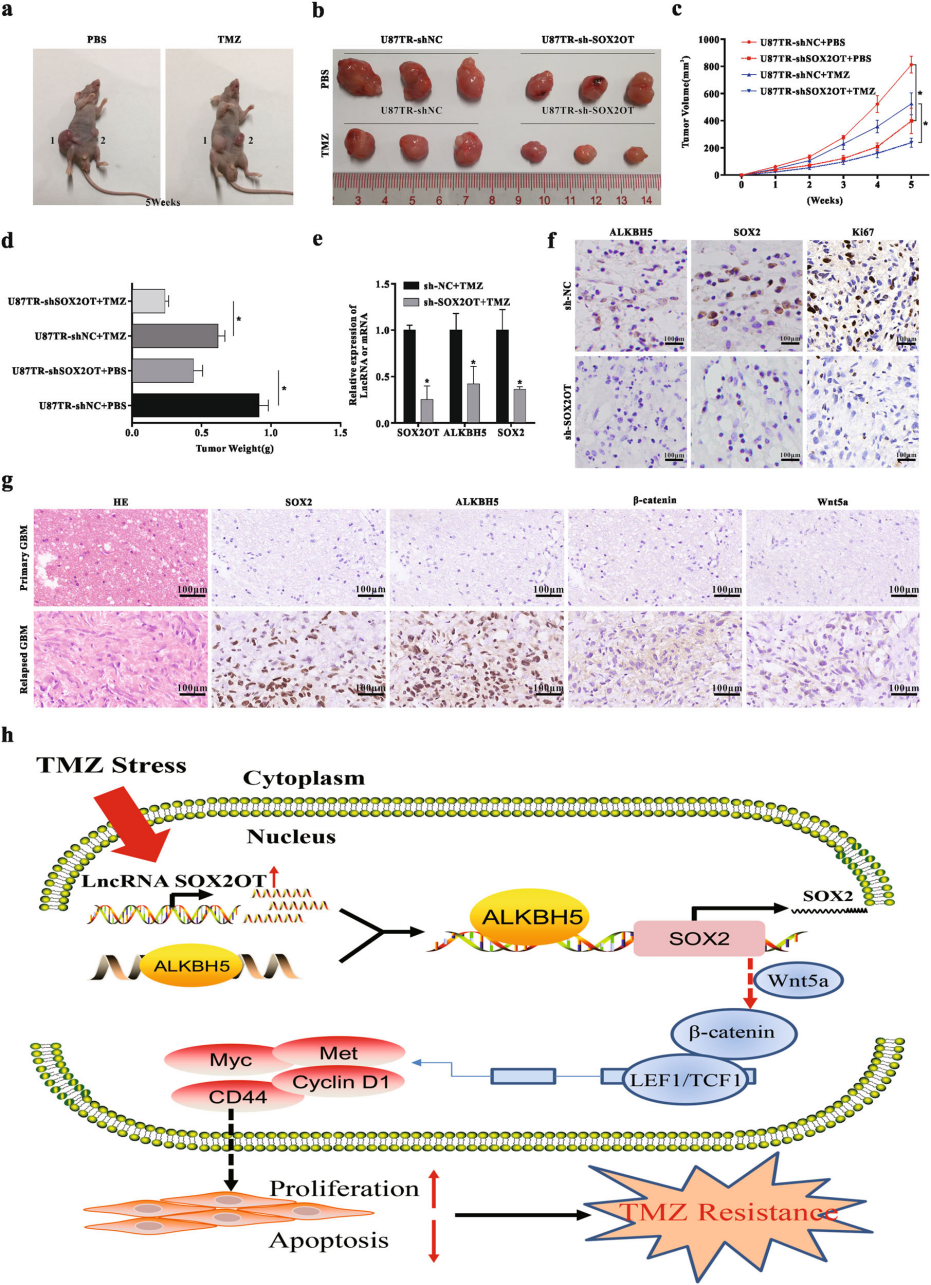
SOX2OT depletion enhances TMZ sensitivity in vivo. **a**–**b** Photographs of tumors that developed in a xenograft-transplanted nude mice tumor model after injection of U87TR-sh-NC (1) or U87TR-sh-SOX2OT (2) cells treated with TMZ (5 μg/g) or PBS at 5 weeks. **c** Growth curve of sh-NC or sh-SOX2OT transfected cell-derived subcutaneous tumor xenografts after TMZ or PBS treatment. *p < 0.05 compared with sh-NC + PBS or sh-NC + TMZ group. **d** Weight of tumor xenografts originating from sh-NC or sh-SOX2OT-transfected cells after treatment with TMZ or PBS at 5 weeks. *p < 0.05 compared with sh-NC + PBS or sh-NC + TMZ group. **e** Relative mRNA expression level of LncRNA SOX2OT, ALKBH5, and SOX2 in U87TR sh-NC or sh-SOX2OT-transfected cell-derived tumor xenografts, *p < 0.05 compared with sh-NC + TMZ group. **f** Immunohistochemistry (IHC) analysis of SOX2, ALKBH5, and Ki67 expression in U87TR-sh-NC or sh-SOX2OT cells-derived tumor xenograft. Scar bar = 100 μm. **g** Immunohistochemistry (IHC) analysis of SOX2, ALKBH5, β-catenin, and Wnt5a in primary GBM and relapsed GBM tissues, respectively. Scar bar = 100 μm. Data are presented as mean ± SD. of three independent experiments. **h** A schematic diagram representing the role of LncRNA SXO2OT in TMZ resistance via upregulating SOX2 expression through ALKBH5-mediated demethylation in GBM cells.

To further assess the effect of SOX2OT on sensitivity to TMZ of U87TR cells in vivo, U87TR-sh-NC or U87TR-sh-SOX2OT cells were synchronously inoculated into the flanks of the immunocompromised nude mice (Fig. 8a). After 5 weeks of injections, U87TR-sh-SOX2OT inoculated mice showed a significantly decreased tumor volume and weight compared with the U87TR-sh-NC-injected group (Fig. 8b–c). In addition, this trend was more significant upon TMZ treatment (Fig. 8d). QRT-PCR and IHC analysis showed that SOX2OT depletion significantly decreased ALKBH5 and SOX2 expression level in resected tumor tissues upon TMZ treatment in comparison with the U87TR-sh-NC group (Fig. 8e–f). Similarly, we verified that SOX2 depletion enhanced TMZ chemosensitivity in vivo (Fig. S6a–c). Finally, we conducted immunohistochemistry of SOX2, ALKBH5, β-catenin, and Wnt5a in primary and relapsed GBM tissues. The expression of SOX2, ALKBH5, β-catenin, and Wnt5a were higher in relapsed GBM specimens compared with primary GBM tissues (Fig. 8g). Meanwhile, SOX2 expression was positively correlated with SOX2OT or ALKBH5 levels in GBM tissues (Fig. S6d–f). Together, these data suggested inhibition of SOX2OT sensitized GBM cells to TMZ in vivo.

## Discussion

The aim of this study was to investigate SOX2OT in TMZ resistance. We first detected SOX2OT in glioma tissues and cell lines and evaluated its clinical relevance. Next, we explored the role of SOX2OT and its potential targeted gene SOX2 on cell growth and apoptosis. Finally, we verified SOX2OT–SOX2 binding of targets and the underlying mechanisms. Figure 8h represents a summary of the results of this study and the suggested mechanism for the role of SOX2OT in TMZ resistance. Overall, these results suggest that increased SOX2OT promotes TMZ resistance by increasing cell proliferation and inhibiting apoptosis. This process involves upregulation of the downstream target SOX2, via ALKBH5 binding the promoter region of the SOX2 gene and increasing its demethylation. SOX2 then increases cell proliferation and decreases apoptosis through the Wnt5a/β-catenin signaling pathway. Importantly, GBM patients with higher SOX2OT expression level had an increased risk of recurrence and worse outcomes compared with patients with lower SOX2OT level. These results indicated that SOX2OT has potential as a marker for prognosis and may be a target to overcome TMZ resistance.

LncRNAs have previously been associated with chemoresistance in GBM. Our previous studies have demonstrated that LncRNA RP11-828N2.4 enhanced TMZ sensitivity by inhibiting miR-10a^28^, and LncRNA AC003092.1 regulated TMZ resistance through the miR-195/TFPI-2-signaling pathway^29^. Moreover, targeted nanocomplex carrying siRNA-MALAT1 sensitized GBM cells to TMZ^30^. In the present study, LncRNAs microarray analysis identified that SOX2OT was significantly overexpressed in U87TR cells compared with the parental U87 cells^17^. Increased SOX2OT expression level conferred chemoresistance, whereas knockdown sensitized GBM cells to TMZ. In addition, we found a positive correlation between SOX2OT and MRPs, including BCRP1, MDR1, MRP1. Besides, previous studies suggested that SOX2OT is involved in maintaining stem cell characteristics^27^. We also demonstrated that overexpressed SOX2OT increased pluripotent transcription factor levels, whereas knocking down its expression decreased them. However, the underlying molecular mechanisms that lead to the markedly differently expression of SOX2OT upon TMZ treatment will need to be further explored in the future.

SOX2OT had a markedly positive correlation with SOX2, a member of SOX family, which was considered as an oncogene in various types of cancer, including glioma^22^. Previous studies have confirmed that SOX2 can promote the lung cancer cell proliferation and can be used as a prognostic indicator^24^. TGF-β induced SOX2 expression and promoted the invasion and metastasis of melanoma cells^31^. In addition, SOX2 is closely correlated with chemoresistance. LncRNA RNALBCS inhibited the self-renewal ability and chemoresistance through epigenetic silencing of SOX2 expression^32^. Similarly, SOX2 induced non-dependent cell proliferation and conferred the resistance to vincristine^33^. As expected, we demonstrated that overexpressed SOX2 also increased MRPs levels, whereas knockdown exerted the opposite effect. Consistently, we found that SOX2OT enhanced the resistance to TMZ by promoting SOX2 expression and inhibiting apoptosis. In addition, our data demonstrated that knockdown of SOX2 increased the expression of proapoptotic proteins under TMZ treatment. This indicated that SOX2 conferred TMZ resistance via inhibiting caspase-dependent apoptosis. Consistently, SOX2 depletion also enhanced TMZ chemosensitivity in vivo.

The RNA FISH assay reveled that SOX2OT was both located in cytoplasm and nuclear in GBM cells. Thus, RBPs may be involved in SOX2OT-regulating SOX2 expression. This study identified ALKBH5 as a putative binding protein of SOX2OT predicted by the LncRNAtor database and confirmed by RNA pulldown assay. Our data also showed that both mRNA and protein level of ALKBH5 were significantly increased in TMZ-resistant cells, which indicates that ALKBH5, an RNA demethylase, may have a vital role in the regulation of TMZ resistance. RNA methylated modification (m^6^A, N^6^-methyladenosine) is the most abundant endogenous RNA modification and is common in eukaryotic mRNAs and LncRNAs^34^. m^6^A dynamically and reversibly methylate the adenine (A) of mRNAs or LncRNAs, which controls RNA stability, location, transportation, shearing, and translation at the post-transcription level^35^. Recent studies have confirmed that m^6^A is closely related to tumors, and can mediate the expression and silencing of genes by regulating the methylation level of RNA, thus causing changes in biological properties^36,37^. Recently, some scholars reported that m^6^A was associated with malignant progression of glioma^38^, such as m^6^A promoted ULK1 protein expression and regulated autophagy process^39^. ALKBH5 directly binds with LncRNA FOXM1-AS to promote FOXM1 expression and maintain the characteristics of glioma stem cells^40^. ALKBH5 inhibited cell migration by reducing the methylation level of LncRNA KCNK15-AS1 in pancreatic cancer^41^. Consistent with the above studies, we also found SOX2 expression was downregulated when ALKBH5 expression was silenced through increasing the methylation level of SOX2. In addition, ALKBH5 was involved in TMZ chemoresistance because the m^6^A levels of total RNAs decreased in TMZ-resistant cells. However, additional studies are needed to fully define the underlying mechanisms.

One pivotal mechanism by which GBM cells become resistant to TMZ is the activation of several signaling pathways. The Wnt/β-catenin signaling pathway is widely involved in the chemotherapy resistance of various malignant tumors^42–44^. Moreover, SOX2 mediates tumor chemoresistance via the Wnt/β-catenin signaling pathway^24^. Hence, we verified that overexpressed SOX2OT activated the Wnt5a/β-catenin signaling pathway through increasing SOX2 expression, and thus upregulated downstream targeted genes including CyclinD1, C-myc, LEF1, TCF1/7, Met/pro-Met.

In summary, we identified that a novel role of SOX2OT in conferring TMZ resistance by interacting with ALKBH5 by reducing the methylation level of SOX2, thus elevating SOX2 expression and activating the Wnt5a/β-catenin signaling pathway to promote cell proliferation and inhibit apoptosis. SOX2OT may be a marker for prognosis of patients with GBM treated with TMZ. But for other grades of glioma, further cell experimental verification is needed, owing to different etiologies and different cellular and molecular structures in low-grade glioma. Moreover, it may also be a potential target for reducing TMZ chemoresistance. However, SOX2OT may exert its effect on TMZ resistance via other signaling pathways, therefore, further elucidating the function of SOX2OT in chemoresistance is important to clarify the mechanisms of TMZ resistance and develop effective therapeutic strategies.

## Materials and methods

### Patients and specimens

All 118 glioma tissues samples and 10 normal samples were obtained from patients who had received surgery and chemotherapy at Zhujiang Hospital (Southern Medical University, Guangzhou, China). These glioma specimens include 85 Grade IV (GBM), 5 Grade III, 13 Grade II, and 15 Grade I astrocytoma cases, and the histologic features of specimens were independently examined by two neuropathologists according to the 2016 WHO criteria for glioma. Specimens were immediately frozen in liquid nitrogen for further protein and RNA extractions after surgical resection. The project protocol was approved by the Ethics Committee of Zhujiang Hospital and written informed consents were obtained from all patients enrolled in this study.

### Cell culture

The human GBM cell lines U87 and U251 were purchased from Cell bank of Chinese Academy of Sciences (Shanghai, China), and it was authenticated and tested for mycoplasma contamination. The TMZ-resistant lines, U87TR and U251TR, were established and maintained in our laboratory^17^. The cells were routinely cultivated in Dulbecco’s modified Eagle’s medium (Invitrogen) containing 10% (v/v) fetal bovine serum (Hyclone, Logan, UT, USA), penicillin (200 units/ml) and streptomycin (100 μg/ml) at 37°C in a 5% CO_2_ humidified air incubator (Thermo Scientific, Waltham, MA, USA). To maintain the TMZ-resistant phenotype, U87TR and U251TR were alternately cultured in TMZ-free medium and medium containing TMZ (200 μg/ml). TMZ was obtained from Sigma (San Francisco, CA, USA) and dissolved in dimethyl sulfoxide (DMSO) with final concentration (50 μg/ml).

### Cell Transfection

For lentiviral transduction, cells were seeded at 50% confluence in six-well cell culture plates and incubated with 1 ml complete medium overnight. Then medium was replaced with 1 ml mixture of OPTI-MEM (Invitrogen, USA) with polybrene (5 μg/ml, Genechem, Shanghai, China). Cells were transfected by adding control shRNA lentiviral vectors, LncRNA SOX2OT shRNA lentiviral vectors, SOX2 shRNA lentiviral vectors, ALKBH5 shRNA lentiviral vectors, Control lentiviral activation vectors, LncRNA SOX2OT lentiviral activation vectors, ALKBH5 lentiviral activation vectors, SOX2 lentiviral activation vectors, respectively. All the lentiviral vectors were obtained from Genechem (Genechem, Shanghai, China). Medium was replaced with complete medium without polybrene 24 h later after transfection. Transduced cells were selected for puromycin (8 μg/ml, sc-108071, Santa Cruz) resistance for 1 week. The gene expression efficiency was detected by qRT-PCR or western blot.

For siRNA transient transfection, 50–100 nmol/L siRNA-NC, siRNA-SOX2OT, siRNA-ALKBH5, siRNA-SOX2, were transfected into GBM cells by using Lipofectamine RNAiMAX Reagent (Thermo Scientific, Waltham, MA, USA) and OPTI-MEM (Invitrogen, USA) according to manufacturer’s protocol. All the RNA oligoribonucleotides were purchased from RiboBio (RiboBio, Guangzhou, China). The target sequences of siRNAs are listed in Supplementary Table S2. The knockdown efficiency was confirmed by qRT-PCR analysis.

### TMZ chemosensitivity and cell viability assay

GBM cells were seeded in 96-well plates and treated with TMZ at different concentrations (0 μg/ml, 100 μg/ml, 200 μg/ml, 300 μg/ml, 400 μg/ml, 500 μg/ml) for 48 h after stable transfection or transient transfection. Followed by incubating with fresh medium contain 10% CCK-8 solution for 2 hours (Dojindo, Kumamoto, Japan), then the absorbance was measured at 450 nm by using Ultra Multifunctional Microplate Reader (Tecan, Switzerland), IC_50_ values were calculated to evaluate the sensitivity to TMZ in the GBM cells. For cell viability assay, GBM cells were exposed to TMZ (50 μg/ml, a quarter of IC_50_ value of the U87 and U251 cells) for 24 h, 48 h, 72 h, and 96 h, then the absorbance was measured at 450 nm after incubating with fresh medium contain 10% CCK-8 solution for 2 hours according to the manufacturer’s instructions.

### RNA isolation, reverse transcription, and quantitative real-time PCR

Total RNA from specimens or cells was isolated by using Trizol Reagent (Takara Bio, Shiga, Japan) according to the manufacturer’s protocol. The quality and yield of the RNA was measured through the absorbance at 260 and 280 nm. First-strand cDNA for LncRNA SOX2OT was generated by using the M-MLV Reverse Transcriptase (Promega, Madison WI, USA). For mRNAs, cDNA was synthesized with the Prime ScriptTM RT reagent kit (Takara Bio, Shiga, Japan). Quantitative real-time PCR assay by using SYBR GREEN PCR Master Mix (Takara Bio, Shiga, Japan) was performed on a 7500 Fast Real-time PCR System (Applied Biosystem, Foster City, CA, USA). The quantitative PCR primers are listed in Supplementary Table S3. U6 snRNA or GAPDH was used as endogenous controls. The relative expression was calculated through relative quantification (2^−△△Ct^), and the data were presented as the mean ± sd of at least three independent experiments.

### Protein extraction and western blot analysis

Total proteins were extracted from specimens and cells using radioimmunoprecipitation assay lysis buffer with protease inhibitor on ice and quantified by bicinchoninic acid protein assay kit (Thermo, USA). The western blot was performed according to standard procedures. The following specific antibodies were applied: ALKBH5 (Millipore Corporation, USA), SOX2, Wnt5a, β-catenin, C-myc, CylinD1, LEF1, TCF1/TCF7, Met/pro-Met, Caspase-3, Caspase-7, Caspase-8, Caspase-9, MDR1, BCRP1, MRP1, and β-actin (Cell Signaling Technology, USA). The antibody information is listed in Supplementary Table S4. The membranes were incubated with HRP-labeled goat-anti-rabbit or goat-anti-mouse secondary antibodies (Cell Signaling Technology, USA). The proteins were detected and visualized by chemiluminescence (Biosciences, Foster City, CA, USA). The protein expression was analyzed by the Image J software, and β -actin was used as loading control.

### Flow cytometric analysis of cell apoptosis

The GBM cells were seeded in six-well plates treated with TMZ (50 μg/ml) for 48 h, then harvested and collected cells for further staining. Cell apoptosis was detected using Annexin-V-FITC Apoptosis Detection Kit (BD Pharmingen, USA) according to the manufacturer’s instructions. Apoptotic rate was analyzed by FACS cytometry (BD Biosciences Inc, Franklin, NJ, USA).

### Fluorescence in situ hybridization

The RNA FISH kit (Roche Applied Science, Germany) was applied to analyze LncRNA subcellular localizations in GBM cells, LncRNA SOX2OT FISH probe and LncRNA 18 S were synthesized by RiboBio Technology Co Ltd (Guangzhou, China), and the assay was performed with the kit according to the manufacture’s protocol. Cells were incubated with 4% paraformaldehyde for 10 min at room temperature, then permeabilized in PBS with 0.5% Triton X-100 on ice for 5 min. Followed by pretreatment with pre-hybridization buffer at 37°C for 30 min. Subsequently, cells were hybridized with 20 μm using Cy3-labeled RNA of LncRNA SOX2OT FISH probe mix in chamber at 37°C for 24 h. Cells were rinsed in 4× SSC with 0.1% Tween-20 for 5 min at 42°C for three times, followed by washing in 2× SSC for 5 min at 42°C and then washed in 1× SSC for 5 min at 42°C. After hybridization, cells were stained with 4′,6-diamidino-2-phenylindole and observed under a fluorescence microscope (Olympus, Tokyo, Japan). The images were analyzed by Image-Pro Plus 6.0 software (Media Cybernetics, Inc, Rockville, MD, USA).

### Immunohistochemistry staining

Immunohistochemistry staining was performed on paraffin-embedded GBM specimens or non-tumor samples according to standard procedures. The samples were incubated with the primary antibodies at 4°C overnight, followed by incubation with biotinylated secondary antibody (1:500 dilutions, Santa Cruz Biotechnology, USA) at room temperature for 2 h. The following primary antibodies were applied: Ki67 (Santa Cruz Biotechnology, USA), SOX2, Wnt5a, β-catenin (Cell Signaling Technology, USA), and ALKBH5 (Millipore Corporation, USA). The location and relative expression of protein were determined through the avidin biotinylated peroxidase complex methods.

### EdU staining assay

The EdU staining assay was performed to assess the GBM cells proliferation with EdU assay Kit (Life Technologies Corporation, Carlsbad, CA, USA) according to manufacturer’s protocol. Cells were cultured in 24-well plates and were incubated for an additional 2 h at 37°C after 10 µm EdU reagent was added into each well. 4% formaldehyde was used to fix the cells for 30 min at room temperature. Followed by washing, EdU was detected with a Click-iT Edu kit at room temperature. The EdU-positive cells were visualized after Hoechst 33342 staining through fluorescent microscope (Olympus, Tokyo, Japan). The ratio of EdU-positive cells was calculated with Image-Pro Plus 6.0 software (Media Cybernetics, Inc., Rockville, MD, USA).

### RNA pulldown and RNA immunoprecipitation assay

Antisense nucleotide probes to the LncRNA SOX2OT sequence were generated to capture LncRNA SOX2OT. RNA probes were transcribed in GBM cells with the MEGAscript T7 Transcription Kit (Ambion, Carlsbad, CA, USA) and labeled with the pierce RNA 3’ End Biotinylation Kit (Life Technology, USA), treated with TURBO DNase (Life Technology, USA), and purified with the RNeasy MiniKit (QIAGEN, Germany). A GBM cell nuclear pellet was resuspended and homogenized with RIP buffer. RNA probes were incubated with the nuclear extract for 1 h at room temperature, and then incubated with Dynabeads Myone Streptavidin C1 (Life Technologies, USA) for 1 h at room temperature. LncRNA SOX2OT associated proteins were identified by western blotting.

The RNA immunoprecipitation assay was performed with EZ-Magna RIP RNA-binding protein immunoprecipitation kit (Millipore Corporation, USA) according to the manufacturer’s recommendations. The magnetic beads coated with 5 μg of normal antibodies against ALKBH5 (Millipore Corporation, USA) were incubated with pre-frozen cell lysates or nuclear extracts overnight at 4°C. The RNA–protein complexes were isolated and washed six times, followed by proteinase K digestion and RNA extraction by TRIzol (Takara, Japan). The relative interaction between ALKBH5 and LncRNA SOX2OT was detected by qPCR and normalized to input. The primers are listed in Supplementary Table S5.

### m^6^A quantification

The EpiQuik m^6^A RNA Methylation Quantification Kit (Colorimetric) (Epigentek, USA) was used for measuring the global m^6^A levels in total RNAs following the manufacturer’s protocol. Total RNA was isolated with TRIzol (Takara, Japan) and treated with deoxyribonuclease I (Sigma, USA). In brief, 200 ng RNAs were coated on assay wells, and the capture antibody solution and detection antibody solution were added to each well separately in a suitable diluted concentration. The m^6^A content was quantified by measuring the absorbance of each well at 450 nm according to the standard curve.

### m^6^A-Seq

m^6^A-IP and library preparation were carried out according to the reported procedure^45^. In brief, poly-A-purified RNA was fragmented with Ambion RNA Fragmentation reagent (Ambion, Carlsbad, CA, USA), and incubated with m^6^A primary antibody at 4°C for 2 h. The mixture was immunoprecipitated through incubation with Protein A beads (Thermo Fisher, MA, USA) at 4°C for 2 h. Followed by washing for three times, the Captured RNA was eluted with m^6^A nucleotide solution and purified with RNA Clean and Concentrator Kit (Zymo, LA, USA). Sequencing was performed on Illumina HiSeq 2000 according to the manufacturer’s recommendations.

### MeRIP-qPCR

The MeRIP-qPCR assay was performed according to a reported protocol^46^. In brief, intact poly-A-purified RNA was denatured to 70°C for 10 min, then transferred on ice and incubated with m^6^A antibody in immunoprecipitation buffer containing RNasin Plus RNase inhibitor (Promega, Madison WI, USA), Tris-HCL, NaCl and lgepal CA-630 (Sigma Aldrich, USA) at 4°C for 2 h. After washing, Dynabeads Protein G (Invitrogen, Carlsbad, CA, USA) were added to the mixture and incubated at 4°C for 2 h with rotation. m^6^A RNA was eluted twice with elution buffer containing N^6^-methyladenosine 5’-monophosphate sodium salt at 4°C for 1 h and precipitated with ethanol at −80°C overnight. The m^6^A RNA was reverse transcribed with random hexamers, and m^6^A enrichment was determined by qRT-PCR analysis. The primers are listed in Supplementary Table S6. Fragmented RNA was directly incubated with m^6^A antibody in immunoprecipitation buffer and performed with the same procedure.

### Electrophoretic mobility-shift assay (EMSA)

EMSA was performed to investigate the association of SOX2 and ALKBH5 according to the protocol by using the chemiluminescent EMSA Kit (Pierce, Rockford, USA). The RNA oligonucleotide probes were synthesized and labeled with biotin in its 5’ terminus (Supplementary Table S7). The incubation of RNA probes and nucleoprotein extract was conducted on ice for 30 min. Then, the mixture was loaded on a 5% gel shift buffer. The probes were transferred electrophoretically to a proper nylon membrane with 380 mA for 25 min by transfer system (BioRad, Hercules, CA, USA). Further, the nylon membrane containing samples was cross-linked by UV-light cross-linker under the following conditions: 254 nm UVC, 120 mJ/cm^2^, 45–60 s. Finally, the results were visualized through the SuperSignal West Femto maximum sensitivity substrate (Thermo Fisher Scientific) and imaged by a Clinx ChemiScope 3400Mini (Science Instrument, Shanghai, China).

### ChIP-PCR assay

The ChIP experiment was performed with the Millipore Magna ChIPA/G kit (Millipore Corporation, USA) according to the manufacturer’s protocol. The antibodies against ALKBH5 were purchased from Cell Signaling Technology (CST, USA). The special primers for site1, site2 and site3 are listed in Supplementary Table S8.

### Tumor xenograft model

To generate murine subcutaneous tumors, 4–5-week-old male BALB/C nude mice were purchased from the Laboratory Animal Center of Southern Medical University (Guangzhou, China), and were housed in a specific pathogen-free facility. The nude mice were grouped randomly without blinding, then 2 × 10^6^ U87TR-sh-NC and U87TR-sh-SOX2OT cells were independently injected subcutaneously into the flanks of nude mice in each group, respectively. When the tumor volume reached 50 mm^3^, the tumor-bearing mice were treated with TMZ (5 μg/g) via intraperitoneal injection (25% final DMSO saline solution, 5 days per week for 3 weeks). Tumor volumes were calculated as the following formula: volume = 0.5 × (length) × (width)^2^. All experimental procedures were performed according to the National Institutes of Health Guide for the Care and Use of Laboratory and were approved by the Animal Experimental Committee of Southern Medical University.

### Statistical analysis

The results were represented as mean ± SD for three independent experiments. Using one-way analysis of variance followed by post hoc Tukey’s test or Student’s *t* test to analysis the data. Kaplan–Meier survival curves were used to evaluate the correlation of LncRNA SOX2OT expression with survival rate. The Mann–Whitney test was applied to assess the significance of difference between groups in tumor specimens. All statistical analyses were conducted with SPSS 19.0 software (SPSS Inc, Chicago, IL, USA) and GraphPad Prism software 7.0 (GraphPad Software, Inc, San Diego, CA, USA).

## Supplementary information

Supplementary Table S1

Supplementary Table S2

Supplementary Table S3

Supplementary Table S4

Supplementary Table S5

Supplementary Table S6

Supplementary Table S7

Supplementary Table S8

Supplementary Figure Legends For CDDis-revised

Figure-S1

Figure-S2

Figure-S3

Figure-S4

Figure-S5

Figure-S6

## References

[1] Cruz-Guilloty F, Perez VL (2011). Molecular medicine: defence against oxidative damage. Nature.

[2] Bowes Rickman C, Farsiu S, Toth CA, Klingeborn M (2013). Dry age-related macular degeneration: mechanisms, therapeutic targets, and imaging. Invest. Ophthalmol. Vis. Sci..

[3] Lopes MBS (2017). The 2017 world health organization classification of tumors of the pituitary gland: a summary. Acta Neuropathol..

[4] Stupp R (2005). Radiotherapy plus concomitant and adjuvant temozolomide for glioblastoma. N. Engl. J. Med..

[5] Thomas A (2017). Temozolomide in the era of precision medicine. Cancer Res..

[6] Lee SY (2016). Temozolomide resistance in glioblastoma multiforme. Genes Dis..

[7] Hombach-Klonisch S (2018). Glioblastoma and chemoresistance to alkylating agents: involvement of apoptosis, autophagy, and unfolded protein response. Pharmacol. Ther..

[8] Messaoudi K, Clavreul A, Lagarce F (2015). Toward an effective strategy in glioblastoma treatment. Part I: resistance mechanisms and strategies to overcome resistance of glioblastoma to temozolomide. Drug Discov. Today.

[9] Erasimus H, Gobin M, Niclou S, Van Dyck E (2016). DNA repair mechanisms and their clinical impact in glioblastoma. Mutat. Res. Rev. Mutat. Res..

[10] Weller M (2015). Mgmt promoter methylation is a strong prognostic biomarker for benefit from dose-intensified temozolomide rechallenge in progressive glioblastoma: the director trial. Clin. Cancer Res..

[11] Huarte M (2015). The emerging role of lncrnas in cancer. Nat. Med..

[12] Engreitz JM (2016). Local regulation of gene expression by lncrna promoters, transcription and splicing. Nature.

[13] Xiong H (2017). Lncrna hulc triggers autophagy via stabilizing sirt1 and attenuates the chemosensitivity of hcc cells. Oncogene.

[14] Ozes AR (2016). Nf-kappab-hotair axis links DNA damage response, chemoresistance and cellular senescence in ovarian cancer. Oncogene.

[15] Li H (2017). Long non-coding rna malat1 decreases the sensitivity of resistant glioblastoma cell lines to temozolomide. Cell. Physiol. Biochem..

[16] Jiang C (2018). Upregulation of casc2 sensitized glioma to temozolomide cytotoxicity through autophagy inhibition by sponging mir-193a-5p and regulating mtor expression. Biomed. Pharmacother..

[17] Zeng H (2017). Genomic profiling of long non-coding rna and mrna expression associated with acquired temozolomide resistance in glioblastoma cells. Int J. Oncol..

[18] Shahryari A, Jazi MS, Samaei NM, Mowla SJ (2015). Long non-coding rna sox2ot: expression signature, splicing patterns, and emerging roles in pluripotency and tumorigenesis. Front. Genet..

[19] Quinn JJ, Chang HY (2016). Unique features of long non-coding rna biogenesis and function. Nat. Rev. Genet..

[20] Wang S (2013). Transient activation of autophagy via sox2-mediated suppression of mtor is an important early step in reprogramming to pluripotency. Cell Stem Cell.

[21] Kamachi Y, Kondoh H (2013). Sox proteins: regulators of cell fate specification and differentiation. Development.

[22] Mansouri S (2016). Sox2: regulation of expression and contribution to brain tumors. CNS Oncol..

[23] Garros-Regulez L (2016). Targeting sox2 as a therapeutic strategy in glioblastoma. Front. Oncol..

[24] He J (2017). Sox2 inhibits wnt-beta-catenin signaling and metastatic potency of cisplatin-resistant lung adenocarcinoma cells. Mol. Med. Rep..

[25] Tripathi SC (2017). Mcam mediates chemoresistance in small-cell lung cancer via the pi3k/akt/sox2 signaling pathway. Cancer Res..

[26] Hou Z (2014). A long noncoding rna sox2ot regulates lung cancer cell proliferation and is a prognostic indicator of poor survival. Int J. Biochem. Cell Biol..

[27] Su R (2017). Knockdown of sox2ot inhibits the malignant biological behaviors of glioblastoma stem cells via up-regulating the expression of mir-194-5p and mir-122. Mol. Cancer.

[28] Liu Y (2016). Long noncoding rna rp11-838n2.4 enhances the cytotoxic effects of temozolomide by inhibiting the functions of mir-10a in glioblastoma cell lines. Oncotarget.

[29] Xu N (2018). Long noncoding rna ac003092.1 promotes temozolomide chemosensitivity through mir-195/tfpi-2 signaling modulation in glioblastoma. Cell Death Dis..

[30] Kim SS (2018). Targeted nanocomplex carrying sirna against malat1 sensitizes glioblastoma to temozolomide. Nucleic Acids Res..

[31] Weina K (2016). Tgf-beta induces sox2 expression in a time-dependent manner in human melanoma cells. Pigment Cell Melanoma Res..

[32] Chen X (2019). Long noncoding rna lbcs inhibits self-renewal and chemoresistance of bladder cancer stem cells through epigenetic silencing of sox2. Clin. Cancer Res..

[33] Choe C (2018). Sox2, a stemness gene, induces progression of nsclc a549 cells toward anchorage-independent growth and chemoresistance to vinblastine. Onco Targets Ther..

[34] Roundtree IA, Evans ME, Pan T, He C (2017). Dynamic rna modifications in gene expression regulation. Cell.

[35] Nilsen TW (2014). Molecular biology. Internal mrna methylation finally finds functions. Science.

[36] Deng X, Su R, Feng X, Wei M, Chen J (2018). Role of n(6)-methyladenosine modification in cancer. Curr. Opin. Genet Dev..

[37] Wang S (2017). Roles of rna methylation by means of n(6)-methyladenosine (m(6)a) in human cancers. Cancer Lett..

[38] Su R (2018). R-2hg exhibits anti-tumor activity by targeting fto/m(6)a/myc/cebpa signaling. Cell.

[39] Jin S (2018). M(6)a rna modification controls autophagy through upregulating ulk1 protein abundance. Cell Res..

[40] Zhang S (2017). M(6)a demethylase alkbh5 maintains tumorigenicity of glioblastoma stem-like cells by sustaining foxm1 expression and cell proliferation program. Cancer Cell.

[41] He Y (2018). Alkbh5 inhibits pancreatic cancer motility by decreasing long non-coding rna kcnk15-as1 methylation. Cell. Physiol. Biochem..

[42] Zhang Z (2018). Inhibition of the wnt/beta-catenin pathway overcomes resistance to enzalutamide in castration-resistant prostate cancer. Cancer Res..

[43] Cai J (2017). Simultaneous overactivation of wnt/beta-catenin and tgfbeta signalling by mir-128-3p confers chemoresistance-associated metastasis in nsclc. Nat. Commun..

[44] Han P (2017). The lncrna crnde promotes colorectal cancer cell proliferation and chemoresistance via mir-181a-5p-mediated regulation of wnt/beta-catenin signaling. Mol. Cancer.

[45] Dominissini D (2012). Topology of the human and mouse m6a rna methylomes revealed by m6a-seq. Nature.

[46] Dominissini D, Moshitch-Moshkovitz S, Salmon-Divon M, Amariglio N, Rechavi G (2013). Transcriptome-wide mapping of n(6)-methyladenosine by m(6)a-seq based on immunocapturing and massively parallel sequencing. Nat. Protoc..

