# Supplementary material for: LncRNA SOX2OT promotes temozolomide resistance by elevating SOX2 expression via ALKBH5-mediated epigenetic regulation in glioblastoma
Source: Cell Death Dis. 2020 May 21;11(5):384. doi: 10.1038/s41419-020-2540-y (PMC7242335; doi:10.1038/s41419-020-2540-y)
Supplement: Supplementary file 1 — Supplementary Table S1 [file 41419_2020_2540_MOESM1_ESM.docx]

**Supplementary Table 1: Association between LncRNA SOX2OT expression levels and clinical/pathological characteristics in 108 patients with glioma**

| Characteristics | No. of patients（n=108） | Low expression  (n=62) | High expression  (n=46) | *P* |
| --- | --- | --- | --- | --- |
| Gender |  |  |  |  |
| Male | 59 (54.6%) | 34（54.8%） | 25（54.3%） | 0.79 |
| Female | 49 (45.4%) | 28（45.2%） | 21（45.7%） |  |
| Age, year |  |  |  |  |
| <50 | 25 (23.1%) | 11（17.7%） | 14（30.4%） | 0.15 |
| ≥50 | 83 (76.9%) | 51（82.3%） | 32（69.6%） |  |
| WHO Grade |  |  |  |  |
| I/II | 28 (25.9%) | 28（45.2%） | 0（0%） | <0.001^*^ |
| III/IV | 80 (74.1%) | 34（54.8%） | 46（100%） |  |

Abbreviation: WHO=World Health Organization.

*Statistical significance (^*^*p*<0.001).
