# Supplementary material for: LncRNA SOX2OT promotes temozolomide resistance by elevating SOX2 expression via ALKBH5-mediated epigenetic regulation in glioblastoma
Source: Cell Death Dis. 2020 May 21;11(5):384. doi: 10.1038/s41419-020-2540-y (PMC7242335; doi:10.1038/s41419-020-2540-y)
Supplement: Supplementary file 2 — Supplementary Table S2 [file 41419_2020_2540_MOESM2_ESM.docx]

Supplementary Table S2: Target sequences of siRNAs used for gene knockdown in GBM cells.

| **siRNA** | **Sequence (5’-3’)** |
| --- | --- |
| LncRNA SOX2OT-siRNA#1 | CAAGACAACACCCTGATCT |
| LncRNA SOX2OT-siRNA#2 | GCCAATCAAACTGCTACAA |
| LncRNA SOX2OT-siRNA#3 | GAACTGCAAGCTCCTTCAA |
| SOX2-siRNA#1 | CCAAGACGCTCATGAAGAA |
| SOX2-siRNA#2 | CCACCTACAGCATGTCCTA |
| SOX2-siRNA#3 | GCTCGCAGACCTACATGAA |
| ALKBH5-siRNA#1 | ACAAGTACTTCTTCGGCGA |
| ALKBH5-siRNA#2 | GCGCCGTCATCAACGACTA |
| ALKBH5-siRNA#3 | CTGAGAACTACTGGCGCAA |
