# Supplementary material for: LncRNA SOX2OT promotes temozolomide resistance by elevating SOX2 expression via ALKBH5-mediated epigenetic regulation in glioblastoma
Source: Cell Death Dis. 2020 May 21;11(5):384. doi: 10.1038/s41419-020-2540-y (PMC7242335; doi:10.1038/s41419-020-2540-y)
Supplement: Supplementary file 3 — Supplementary Table S3 [file 41419_2020_2540_MOESM3_ESM.docx]

Supplementary Table S3: The primers used in qRT-PCR analysis in GBM cells.

| **LncRNA/mRNA** | **Forward primer** | **Reverse primer** |
| --- | --- | --- |
| LncRNA SOX2OT | TTAGCAGCAAACCCAGAAC | TGCTGAGGATACATGACGAA |
| U6 | CTCGCTTCGGCAGCACA | AACGCTTCACGAATTTGCGT |
| SOX2 | GCAGAGAAGAGAGTGTTTGC | GAGAGGCAAACTGGAATCA |
| MDR1 | GGGATGGTCAGTGTTGATGGA | GCTATCGTGGTGGCAAACAATA |
| BCRP1 | ATGTCACGTGGAATACCAGC | GAAGACTGAACTCCCTTCCT |
| MRP1 | ATGTCACGTGGAATACCAGC | GAAGACTGAACTGCAGA |
| DKK1 | CCTTGAACTCGGTTCTCAATTCC | CAATGGTCTGGTACTTATTCCCG |
| Wnt3a | AGCTACCCGATCTGGTGGTC | CAAACTCGATGTCCTCGCTAC |
| Wnt5a | GCCAGTATCAATTCCGACATCG | TCACCGCGTATGTGAAGGC |
| β-catenin | AAAGCGGCTGTTAGTCACTGG | CGAGTCATTGCATACTGTCCAT |
| AXIN2 | CAACACCAGGCGGAACGAA | GCCCAATAAGGAGTGTAAGGACT |
| FZD4 | CCTCGGCTACAACGTGACC | TGCACATTGGCACATAAACAGA |
| C-myc | GGCTCCTGGCAAAAGGTCA | CTGCGTAGTTGTGCTGATGT |
| ALKBH5 | CGGCGAAGGCTACACTTACG | CCACCAGCTTTTGGATCACCA |
| CD15 | TTGGGACCTCCTAGTTCCAC | TGTAAGGAAGCCACATTGGA |
| CD133 | CAGGTAAGAACCCGGATCAA | TCAGATCTGTGAACGCCTTG |
| Nestin | GCAGCAGGAAATATGGGAAG | TCTCATGGCTCTGGTTTTCC |
| OCT-4 | GGGAGATTGATAACTGGTGTGTT | GTGTATATCCCAGGGTGATCCTC |
| WTAP | TTGTAATGCGACTAGCAACCAA | GCTGGGTCTACCATTGTTGATCT |
| YTHDF1 | ACCTGTCCAGCTATTACCCG | TGGTGAGGTATGGAATCGGAG |
| YTHDF2 | AGCCCCACTTCCTACCAGATG | TGAGAACTGTTATTTCCCCATGC |
| YTHDF3 | GCTATCCACCTAGTTCTCTTGGG | ATGCCAGGCACCTTACTCAAA |
| FTO | ACTTGGCTCCCTTATCTGACC | TGTGCAGTGTGAGAAAGGCTT |
| METTL3 | AGATGGGGTAGAAAGCCTCCT | TGGTCAGCATAGGTTACAAGAGT |
| METTL14 | GTTGGAACATGGATAGCCGC | CAATGCTGTCGGCACTTTCA |
| GAPDH | GAGGTGATAGCATTGCTTTCG | CAAGTCAGTGTACAGGTAAGC |
