# Supplementary material for: LncRNA SOX2OT promotes temozolomide resistance by elevating SOX2 expression via ALKBH5-mediated epigenetic regulation in glioblastoma
Source: Cell Death Dis. 2020 May 21;11(5):384. doi: 10.1038/s41419-020-2540-y (PMC7242335; doi:10.1038/s41419-020-2540-y)
Supplement: Supplementary file 4 — Supplementary Table S4 [file 41419_2020_2540_MOESM4_ESM.docx]

Supplementary Table S4: Information on antibodies used in GBM cells.

| Reagent | Source | Identifier |
| --- | --- | --- |
| Anti-SOX2 | Cell Signaling Technology | Cat#3579 |
| Anti-Wnt5a | Cell Signaling Technology | Cat#2530 |
| Anti-β-catenin | Cell Signaling Technology | Cat#8480 |
| Anti-C-myc | Cell Signaling Technology | Cat#5605 |
| Anti-CyclinD1 | Cell Signaling Technology | Cat#2978 |
| Anti-LEF1 | Cell Signaling Technology | Cat#2230 |
| Anti-TCF1/TCF7 | Cell Signaling Technology | Cat#2203 |
| Anti-Met/pro-Met | Cell Signaling Technology | Cat#8198 |
| Anti-Caspase-3 | Cell Signaling Technology | Cat#9662 |
| Anti-Caspase-7 | Cell Signaling Technology | Cat#9492 |
| Anti-Caspase-8 | Cell Signaling Technology | Cat#9746 |
| Anti-Caspase-9 | Cell Signaling Technology | Cat#9504 |
| Anti-ALKBH5 | Millipore | Cat#ABE547 |
| Anti-β-actin | Cell Signaling Technology | Cat#4970 |
| Anti-Ki67 | Santa Cruz Biotechnology | Cat#sc-15402 |
| Anti-MDR1 | Cell Signaling Technology | Cat#13342 |
| Anti-BCRP1 | Cell Signaling Technology | Cat#42078 |
| Anti-MRP1 | Cell Signaling Technology | Cat#14685 |
