# Supplementary material for: LncRNA SOX2OT promotes temozolomide resistance by elevating SOX2 expression via ALKBH5-mediated epigenetic regulation in glioblastoma
Source: Cell Death Dis. 2020 May 21;11(5):384. doi: 10.1038/s41419-020-2540-y (PMC7242335; doi:10.1038/s41419-020-2540-y)
Supplement: Supplementary file 5 — Supplementary Table S5 [file 41419_2020_2540_MOESM5_ESM.docx]

Supplementary Table S5: The special primers for RIP-PCR in GBM cells.

| Name | Forward | Reverse |
| --- | --- | --- |
| SOX2OT-1 | tccatggaatgaatgaaatgttctc | ccagtctttccatcagcctcc |
| SOX2OT-2 | ccttgcaccagggctgac | tgggactttccttatcagagtatcaa |
| SOX2OT-3 | gattgcagtggcaaagctagg | caccggacagaaccagttgaa |
| U1(control) | gggagataccatgatcacgaaggt | ccacaaattatgcagtcgagtttccc |
