# Supplementary material for: LncRNA SOX2OT promotes temozolomide resistance by elevating SOX2 expression via ALKBH5-mediated epigenetic regulation in glioblastoma
Source: Cell Death Dis. 2020 May 21;11(5):384. doi: 10.1038/s41419-020-2540-y (PMC7242335; doi:10.1038/s41419-020-2540-y)
Supplement: Supplementary file 6 — Supplementary Table S6 [file 41419_2020_2540_MOESM6_ESM.docx]

Supplementary Table S6: The primers used in MeRIP-PCR analysis in GBM cells.

| **Peak** | **Forward primer** | **Reverse primer** |
| --- | --- | --- |
| hsa-MACS_peak_61196A | AGAGAAGTTTGAGCCCCAGG | CGCTTCCCTCCTCCTCTG |
| hsa-MACS_peak_61196B1 | GTCAAGCGGCCCATGAATG | GTTGTGCATCTTGGGGTTCT |
| hsa-MACS_peak_61196B2 | GCGCTGCACATGAAGGAG | GGGCAGCGTGTACTTATCCT |
| hsa-MACS_peak_61196C | GACAGTTACGCGCACATGAA | AGCCGTTCATGTAGGTCTGC |
| hsa-MACS_peak_61196D1 | TACCTCTTCCTCCCACTCCA | CCGGGGAGATACATGCTGAT |
| hsa-MACS_peak_61196D2 | GAAATGGGAGGGGTGCAAAA | TACCGGGTTTTCTCCATGCT |

Peak Site1:

**AGAGAAGTTTGAGCCCCAGG**CTTAAGCCTTTCCAAAAAATAATAATAACAATCATCGGCGGCGGCAGGATCGGC**CAGAGGAGGAGGGAAGCG**

Peak Site2:

**GTCAAGCGGCCCATGAATG**CCTTCATGGTGTGGTCCCGCGGGCAGCGGCGCAAGATGGCCCAGG**AGAACCCCAAGATGCACAAC**

Peak Site3:

**GCGCTGCACATGAAGGAG**CACCCGGATTATAAATACCGGCCCCGGCGGAAAACCAAGACGCTCATGAAGA**AGGATAAGTACACGCTGCCC**

Peak Site4:

**GACAGTTACGCGCACATGAA**CGGCTGGAGCAACGGCAGCTACAGCATGATGCAGGACCAGCTGGGCTACCCGCAGCACCCGGGCCTCAATGCGCACGGCGCAGCGCAGATGCAGCCCATGCACCGCTACGACGTGAGCGCCCTGCAGTACAACTCCATGACCAGCTC**GCAGACCTACATGAACGGCT**

Peak Site5:

**TACCTCTTCCTCCCACTCCA**GGGCGCCCTGCCAGGCCGGGGACCTCCGGGACATG**ATCAGCATGTATCTCCCCGG**

Peak Site6:

GA**AATGGGAGGGGTGCAAAA**GAGGAGAGTAAGAAAC**AGCATGGAGAAAACCCGGTA**
