# Supplementary material for: LncRNA SOX2OT promotes temozolomide resistance by elevating SOX2 expression via ALKBH5-mediated epigenetic regulation in glioblastoma
Source: Cell Death Dis. 2020 May 21;11(5):384. doi: 10.1038/s41419-020-2540-y (PMC7242335; doi:10.1038/s41419-020-2540-y)
Supplement: Supplementary file 7 — Supplementary Table S7 [file 41419_2020_2540_MOESM7_ESM.docx]

Supplementary Table S7: The probe sequences used in RNA EMSA assay in GBM cells.

| Name | Probe Sequence |
| --- | --- |
| SOX2 | tgcgcggcctgggctttgcggccaccacaatggaaatctacggggaaaatgccagggctggttctgctgg |
| Label | ccccgtagatttccattgtggtggccgcaaagcccaggccgcgca |
