# Supplementary material for: LncRNA SOX2OT promotes temozolomide resistance by elevating SOX2 expression via ALKBH5-mediated epigenetic regulation in glioblastoma
Source: Cell Death Dis. 2020 May 21;11(5):384. doi: 10.1038/s41419-020-2540-y (PMC7242335; doi:10.1038/s41419-020-2540-y)
Supplement: Supplementary file 8 — Supplementary Table S8 [file 41419_2020_2540_MOESM8_ESM.docx]

Supplementary Table S8: The primers used in ChIP-qPCR assay in GBM cells.

| Name | site | Forward | Reverse |
| --- | --- | --- | --- |
| SOX2 | 1 | atgccaggaagttgaaatcacc | cgtagcaaaggggatgcttct |
| SOX2 | 2 | ctctagagtctgccttatggtccg | cttccgctctcctctcttacctta |
| SOX2 | 3 | cagtaacaggctagggagggc | cctggtctacccttactcaccctaa |
| GAPDH promoter | 1 | catgggtgtgaaccatgaga | gtcttctgggtggcagtgat |

Predicted binding sites:

Site1

>chr3:181710812-181711111

ACGGGGAAAATGCCAGGGCTGGTTCTGCTGGAGTCCTGGGAACTCTGCGTGGGAGGGAGTTTGTGACTGCGGCCCAAAAGCCACCTCCATACAGTGCCGTGGGATGCCAGGAAGTTGAAATCACCCTCCCCCATCGCCTGCACTTTTGAGCGCCCTTCCGTCTGTGTCTTTCCCCAGCCCCCATTTGAAAGCCGCACGACCGAAACCCTTCTTACGGGGAGGCATGGGATGGGAATGGGGAGTGGGGGCAGACAGTAGAAGCATCCCCTTTGCTACGGTTGAATGAAGACAGTCTAGTGG

Site2

>chr3:181711112-181711472

GAGATGTGGCTGGGGCTAAGAGGAAGAGCTGCAGTTTCCTGGGCCAAAGAGCTGAGTTGGACAGGGAGATGGCAGCTTACCAAGGCCTGCTGGTTCTCAGCTCTAGAGTCTGCCTTATGGTCCGAGCAGGATTTATTTTTAAGAACAGAGCAAGTTACGTGGAAGCAAGGAAGGTTTTGAGGACAGAGGTTTGGGTCTCCTAACTTCTAGTCGGGACTGTGAGAAGGGCGTGAGAGAGTGTTGGCACCTGTAAGGTAAGAGAGGAGAGCGGAAGAGCGCAGTACGGGAGCGGCACCAGAGGGGCTGGAGTTGGGGGGGAGTGCTGTGGATGAGCGGGAGAACAATGACACACCAACTCCTG

Site3

>chr3:181709973-181710330

GGCAGGAAGGTTGATTGGAAATAACTTAAGGAAAGTCTGCAGAATTCTTTTTTTTACAACTTTTCTGAGTTTCCAGTGGGTATATTTAGTGTGAGTTTGACAGTAACAGGCTAGGGAGGGCAGAGATTGGAGAAATTGGGGGTCGGGGGAGTGATTATGGGAAGAAGGTTAGTAAGGAACAAAACAATGCACCGTTTTGTAAAGATAATAAATGGAACGTGGCTGGTAGATACTATTCAGTACATTTTCTTAGGGTGAGTAAGGGTAGACCAGGGGAGGAGGGGGCGGAGAGAGTGTTACAGAAGAAAGAAAATAAGTAACCCTGATGGTTTAAGCCCTTTATAAAAAAGAAATGGCA
