# Supplementary material for: LncRNA SOX2OT promotes temozolomide resistance by elevating SOX2 expression via ALKBH5-mediated epigenetic regulation in glioblastoma
Source: Cell Death Dis. 2020 May 21;11(5):384. doi: 10.1038/s41419-020-2540-y (PMC7242335; doi:10.1038/s41419-020-2540-y)
Supplement: Supplementary file 9 — Supplementary Figure Legends For CDDis-revised [file 41419_2020_2540_MOESM9_ESM.docx]

**Supplementary Fig S1, related to Fig 2. SOX2OT upregulation confers TMZ resistance, facilitates cell proliferation and inhibits cell apoptosis in GBM cells.** (**a**) qRT-PCR analysis ofSOX2OT expression in U87TR and U251TR cells transfected with siRNAs. **p*<0.05 compared with si-NC group (**b**) qRT-PCR analysis of LncRNA SOX2OT expression or in U87 and U251 cells transfected with lentivirus vectors. **p*<0.05 compared with LV-NC group (**c**) The TMZ sensitivity of U87TR and U251TR cells after sh-NC or sh- SOX2OT transfection measured by CCK-8 assay. ^*^*p* <0.05 compared with sh-NC group cells. (**d**) The TMZ sensitivity of U87 and U251 Cells after LV-NC or LV-SOX2OT transfection measured by CCK-8 assay. **p*<0.05 compared with LV-NC group cells. (**e-g**) The rate of apoptosis of sh-SOX2OT or LV-SOX2OT cells without TMZ treatment was determined by the Annexin-V/FITC double staining. ^*^*p* <0.05 compared with sh-NC or LV-NC group cells. (**h-j**) The qRT-PCR and western blot analysis of chemoresistance-related proteins in TMZ resistant cells with sh-NC or sh-SOX2OT transfection. ^*^*p* <0.05 compared with sh-NC group (**k-m**) The qRT-PCR and western blot analysis of chemoresistance-related proteins in the parental cells with LV-NC or LV-SOX2OT transfection. ^*^*p* <0.05 compared with LV-NC group (**n-o**) The qRT-PCR analysis of pluripotent transcription factors in TMZ-resistant cells after sh-NC or sh-SOX2OT transfection. ^*^*p* <0.05 compared with sh-NC group (**p-q**) The mRNA expression of pluripotent transcription factors in LV-NC or LV-SOX2OT transfected cells. ^*^*p* <0.05 compared with LV-NC group. Data are presented as mean ± SD. of three independent experiments.

**Supplementary Fig S2, related to Fig 4. SOX2OT binds with RNA demethylase ALKBH5, which is involved in TMZ resistance.** (**a**) Cellular localization of lncRNA SOX2OT in U87, U251 cells by RNA-FISH assay. Scar bar = 50μm. (**b**)The potential RNA binding proteins combined with LncRNA SOX2OT were predicted by the LncRNAtor database (http://lncrnator.ewha.ac.kr/index.htm). (**c-d**) The mRNA expression analysis of m^6^A related genes between TMZ resistant cells and its parented cells. ^*^*p* <0.05 compared with the parented cells. (**e-f**) The mRNA expression analysis of m^6^A related genes in parented cells under different concentrations TMZ treated. (**g-h**) The qRT-PCR and western blot analysis of ALKBH5 expression in U87TR and U251TR cells transfected with siRNAs and sh-ALKBH5 transfected lentivirus vectors. ^*^*p* <0.05 compared with si-NC group. (**i-j**) The qRT-PCR and western blot analysis of ALKBH5 expression in U87 and U251 cells transfected with lentivirus vectors. ^*^*p* <0.05 compared with LV-NC group.

**Supplementary Fig S3, related to Fig 6.** **SOX2OT regulates TMZ resistance through SOX2.** (**a**) The qRT-PCR and western blot analysis of SOX2 expression in U87TR and U251TR cells transfected with siRNAs. ^*^*p* <0.05 compared with si-NC group. (**b)** The qRT-PCR and western blot analysis of SOX2 expression in U87 and U251 cells transfected with lentivirus vectors. ^*^*p* <0.05 compared with LV-NC group. (**c**) The TMZ sensitivity of U87TR and U251TR cells upon sh-NC or sh-SOX2 transfection through CCK8-assay. **p*<0.05 compared with sh-NC group cells. (**d**) The TMZ sensitivity of U87 and U251 cells upon LV-SOX2 transfection measured by CCK8-assay. **p*<0.05 compared with LV-NC group cells. (**e-g**) The qRT-PCR and western blot analysis of chemoresistance-related proteins in TMZ resistant cells with sh-NC or sh-SOX2 transfection. **p*<0.05 compared with sh-NC group cells. (**h-j**) The qRT-PCR and western blot analysis of chemoresistance-related proteins in the parental cells with LV-NC or LV-SOX2 transfection. **p*<0.05 compared with LV-NC group cells. (**k**) The TMZ sensitivity of TMZ-resistant cells with sh-SOX2OT and LV-SOX2 co-transfection was measured by CCK-8 assay. **p*<0.05 compared with sh-SOX2OT group cells. **(l)** The cell viability of TMZ-resistant cells after sh-SOX2OT and LV-SOX2 co-transfection was measured by CCK8-assay. **p*<0.05 compared with sh-SOX2OT group cells. (**m**) The TMZ sensitivity of parental cells with LV-SOX2OT and sh-SOX2 co-transfection was measured by CCK-8 assay. **p*<0.05 compared with LV-SOX2OT group cells. **(n)** The cell viability of U87 and U251 cells with co-transfected LV-SOX2OT and sh-SOX2 was measured by CCK8-assay. **p*<0.05 compared with LV-SOX2OT group cells. (o-q) The Rate of apoptosis of sh-SOX2 or LV-SOX2 cells treated with TMZ (50μg/ml) for 48h was determined by the flow cytometry. **p* <0.05 compared with sh-NC or LV-NC group cells.

**Supplementary Fig S4, related to Fig 6. SOX2OT regulates TMZ resistance through SOX2.** (**a-c**) The rate of apoptosis of sh-SOX2 or LV-SOX2 cells without TMZ treatment was determined by the flow cytometry. **p* <0.05 compared with sh-NC or LV-NC group cells. (**d**) The rate of apoptosis of U87TR and U251TR cells with sh-SOX2OT and LV-SOX2 co-transfection without TMZ treatment measured by flow cytometry analysis. **p*<0.05 compared with sh-SOX2OT group cells. (**e)** The rate of apoptosis of U87 and U251 cells with LV-SOX2OT and sh-SOX2 co-transfection without TMZ treatment measured by flow cytometry analysis. **p*<0.05 compared with LV-SOX2OT group cells. (**f)** The statistics of apoptosis rate in sh-SOX2OT and LV-SOX2 co-transfection or LV-SOX2OT and sh-SOX2 co-transfection cells. **(g-i)** The Rate of EdU cells in sh-SOX2 or LV-SOX2 transfected cells treated with TMZ (50μg/ml) for 48h was determined by EdU staining. ^*^*p* <0.05 compared with sh-NC or LV-NC group cells. Scar bar = 50μm. Data are presented as mean ± SD. of three independent experiments.

**Supplementary Fig S5, related to Fig 7. The Wnt5a/β-catenin signaling Pathway is involved in SOX2OT regulating SOX2 in TMZ resistance.** (**a-b**) qRT-PCR analysis for Wnt/β-catenin signaling pathway related gene mRNAs in TMZ-resistant cells. ^*^*p* <0.05 compared with the parented cells. (**c-d**) Western blot analysis for β-catenin, wnt5a protein in glioma cells with different concentrations of TMZ. (**e**) Western blot analysis for Wnt/β-catenin signaling pathway related genes in U87 and U251 cells with LV-SOX2OT and sh-SOX2 co-transfection. (**f**) Western blot analysis for Wnt/β-catenin signaling pathway related genes in U87TR and U251TR cells with sh-SOX2OT and LV-SOX2 co-transfection.

**Supplementary Fig S6. SOX2 depletion enhances TMZ sensitivity in vivo.** (**a**) Photographs of tumors that developed in xenograft-transplanted nude mice tumor model after injection of U87TR-sh-NC (1) or U87TR-sh-SOX2 (2) Cells treated with TMZ (5μg/g) or PBS at 5 weeks. (**b**) Growth curve of sh-NC or sh-SOX2 cells-derived subcutaneous tumor xenografts after TMZ or PBS treated. **p*<0.05 compared with sh-NC+TMZ or sh-NC+PBS group. **(c)** Weight of tumor xenografts originated from sh-NC or sh-SOX2 cells after treatment of TMZ or PBS at 5 weeks. **p*<0.05 compared with sh-NC +TMZ or sh-NC+PBS group. **(d)** IHC scores of SOX2 in primary and relapsed GBM tissues. **P*<0.05 compared with primary GBM tissues. **(e-f)** The correlation analysis of SOX2 IHC scores and SOX2OT (e) or ALKBH5 scores (f) in GBM tissues. Data are presented as mean ± SD of three independent experiments.
